# Differential Roles for DUSP Family Members in Epithelial-to-Mesenchymal Transition and Cancer Stem Cell Regulation in Breast Cancer

**DOI:** 10.1371/journal.pone.0148065

**Published:** 2016-02-09

**Authors:** Tara Boulding, Fan Wu, Robert McCuaig, Jennifer Dunn, Christopher R. Sutton, Kristine Hardy, Wenjuan Tu, Amanda Bullman, Desmond Yip, Jane E. Dahlstrom, Sudha Rao

**Affiliations:** 1 Health Research Institute, Faculty of ESTeM, University of Canberra, Bruce, ACT, 2617, Australia; 2 Anatomical Pathology, ACT Pathology, The Canberra Hospital, Garran ACT, 2605, Australia; 3 ANU Medical School, Australian National University, Acton, ACT, 2601, Australia; 4 Department of Medical Oncology, The Canberra Hospital, ACT, Garran, 2605 Australia; Wayne State University School of Medicine, UNITED STATES

## Abstract

Dual-specificity phosphatases (DUSPs) dephosphorylate threonine/serine and tyrosine residues on their substrates. Here we show that DUSP1, DUSP4, and DUSP6 are involved in epithelial-to-mesenchymal transition (EMT) and breast cancer stem cell (CSC) regulation. DUSP1, DUSP4, and DUSP6 are induced during EMT in a PKC pathway signal-mediated EMT model. We show for the first time that the key chromatin-associated kinase PKC-θ directly regulates a subset of DUSP family members. DUSP1, DUSP4, and DUSP6 globally but differentially co-exist with enhancer and permissive active histone post-translational modifications, suggesting that they play distinct roles in gene regulation in EMT/CSCs. We show that nuclear DUSP4 associates with the key acetyltransferase p300 in the context of the chromatin template and dynamically regulates the interplay between two key phosphorylation marks: the 1834 (active) and 89 (inhibitory) residues central to p300’s acetyltransferase activity. Furthermore, knockdown with small-interfering RNAs (siRNAs) shows that DUSP4 is required for maintaining H3K27ac, a mark mediated by p300. DUSP1, DUSP4, and DUSP6 knockdown with siRNAs shows that they participate in the formation of CD44^hi^/CD24^lo^/EpCAM^+^ breast CSCs: DUSP1 knockdown reduces CSC formation, while DUSP4 and DUSP6 knockdown enhance CSC formation. Moreover, DUSP6 is overexpressed in patient-derived HER2^+^ breast carcinomas compared to benign mammary tissue. Taken together, these findings illustrate novel pleiotropic roles for DUSP family members in EMT and CSC regulation in breast cancer.

## Introduction

Breast cancer is the most common malignancy in women worldwide [1]. Although chemotherapy and radiotherapy benefit women and improve patient survival, some cancers are treatment resistant [2]. Epithelial-to-mesenchymal transition (EMT) is a biological program in which epithelial cells lose cell-cell junctions, apical-basal polarity, and acquire an invasive mesenchymal phenotype [3]. EMT has been implicated in cancer initiation, progression, metastasis, resistance to conventional therapies, and recurrence [4]. This process is induced via complex interactions between extracellular signals and factors that activate downstream signalling pathways including, but not limited to, the WNT, TGF-β, Notch, Hedgehog, PI3-kinase/AKT, and mitogen-activated protein kinase (MAPK) pathways [5]. These pathways activate EMT-inducing transcription factors (EMT-TFs) such as Snail and Slug, which directly regulate inducible gene expression [6,7].

EMT can induce the formation of a small subpopulation of cancer stem cells (CSCs) and endow these cells with stem cell-like properties including the capacity to self-renew and differentiate [8–10]. CSCs play a pivotal role in metastasis, relapse, and resistance to standard anti-cancer therapies. Breast CSCs display a CD44^+^/CD24^-^ cell surface marker profile and are known to form a subpopulation of circulating tumour cells [10–12]. Breast CSCs are also enriched after cytotoxic therapy *in vitro*, *in vivo*, and in patients, thus implicating these cells in disease recurrence and metastasis [13].

Dual-specificity phosphatases (DUSPs) belong to a protein family responsible for dephosphorylating threonine/serine and tyrosine residues on their substrates. DUSPs selectively dephosphorylate the signalling MAPKs, implicating them in signal transduction. Several DUSP family members are thought to be involved in breast cancer metastasis including DUSP1, DUSP4, and DUSP6. DUSP1 is overexpressed in breast carcinomas compared to normal mammary tissue, with greater expression in infiltrating carcinomas than *in situ* carcinomas [14–16]. Moreover, DUSP1 is exclusively expressed in HER2^+^ carcinomas, which are relatively poor prognosis tumours but amenable to HER2-targeting therapies, and DUSP1 expression is associated with an increased risk of metastasis and shorter overall survival [17]. In contrast, DUSP4 acts as a tumour suppressor, with low expression associated with increased tumour grade, recurrence, and poor prognosis in breast cancer patients [18,19]. However, DUSP4 has also been shown to be upregulated in malignant tissues [16,20]. Similar to DUSP1, DUSP6 is upregulated in HER2^+^ carcinomas; however, little is known about its expression in normal mammary tissue [21,22]. Furthermore, DUSP1 expression is associated with resistance to cytotoxic chemotherapies including mechlorethamine, doxorubicin, paclitaxel, and cyclophosphamide [23,24] and resistance to radiotherapy [17]. Similarly, DUSP4 is implicated in doxorubicin and cisplatin chemoresistance [25,26]. It has also been suggested that DUSP6 overexpression may confer resistance to the commonly used hormone therapy drug, tamoxifen [27].

However, little is known about how DUSPs regulate EMT and CSCs in breast cancer. DUSP1 knockdown reduces survival of HER2^+^/CD44^+^/CD24^-^ breast CSCs and sensitises them to irradiation [17], suggesting a role for DUSP1 in HER2^+^/CD44^+^/CD24^-^ breast CSC survival and the radiotherapy-resistant phenotype. Treatment of MCF-7 breast cancer cells with doxorubicin can induce EMT, and DUSP4 knockdown partially abrogates this effect. Moreover, specific DUSP4 overexpression in MCF-7 cells can increase mesenchymal protein expression and decrease epithelial protein expression [25]. Overall, these studies implicate DUSP4 as an attractive candidate EMT regulator.

How DUSP family members regulate EMT and breast CSC formation and maintenance remains unknown. Here we show that DUSP1, DUSP4, and DUSP6 are induced during EMT and are involved in forming and maintaining breast CSCs. DUSP1, DUSP4, and DUSP6 globally but differentially co-exist with enhancer and permissive active histone post-translational modifications, suggesting that they play distinct roles in gene regulation in EMT/CSCs. We show that nuclear DUSP4 associates with the key acetyltransferase p300 in the context of the chromatin template and dynamically regulates the interplay between two key phosphorylation marks: the 1834 and 89 residues, which are critical for the histone acetyltransferase activity of p300. These events are abolished by pan-PKC and PKC-θ-selective inhibitors, suggesting a key role for the PKC-θ pathway in this novel molecular mechanism operating in the context of EMT in breast cancer. Knockdown with small-interfering RNAs (siRNAs) shows that DUSP4 is required for H3K27ac, a mark mediated by p300. Importantly, we show that the chromatin-associated kinase PKC-θ directly regulates specific DUSP family members. This is the first report of crosstalk between nuclear kinases and phosphatases in the epigenomic context in breast EMT. Overall, based on these novel results, we propose that nuclear DUSPs mark the EMT and CSC epigenome at PKC-targeted gene loci in breast cancer.

## Materials and Methods

### Cell culture

MCF-7 and MDA-MB-231 cells were obtained from the American Type Culture Collection (Manassas, VA). Cells were cultured in DMEM (Invitrogen, Life Technologies, Carlsbad, CA) supplemented with 10% FBS, 2mM L-glutamine, and 1% penicillin-streptomycin-neomycin. MCF-7 cells were stimulated with 1.32 ng/ml phorbol 12-myristate 13-acetate (PMA) (Sigma-Aldrich, St Louis, MO) or 5 ng/ml recombinant TGF-β1 (R&D Systems, Minneapolis, MN) for 60 h, as previously described [28]. 0.65 nM triptolide (Santa Cruz, Dallas, TX) and 1 μM NSC 95397 (Santa Cruz) were used for inhibitor experiments. Forward transfection reactions were performed for 6 h with 50 nM human DUSP1 siRNA (sc-35937), DUSP4 siRNA (sc-38998), DUSP6 siRNA (sc-39000), 20 nM PKC-θ siRNA (sc-36252), and 10 nM MOCK siRNA (sc-36869) (Santa Cruz) using Lipofectamine 2000 (Invitrogen).

### Cell migration/invasion assay

Cells were cultured in serum-free DMEM overnight, detached by scraping, and centrifuged at 500 x *g* for 10 min at room temperature. 1×10^4^ cells were counted and transferred into Falcon BioCoat Matrigel Invasion Chambers with 8.0 μm PET membranes (FAL354481; Corning Inc., Cornings, NY) or Falcon BioCoat Control Inserts with 8.0 μm PET membranes (FAL354578; Corning) in 100 μl serum-free medium. Complete serum-positive DMEM was added to the bottom layer to promote chemotaxis. Thereafter, plates were cultured in a humidified incubator at 37°C for 24 h. Chambers or control inserts were washed twice with ddH_2_O prior to staining with 0.01% crystal violet (dissolved in DPBS, Sigma) for 10 min. Stained chambers or control inserts were photographed at x 40 magnification using a Leica confocal microscope and purple-staining spots counted manually. Percentage efficiency of migration/invasion was calculated using the equation: (no. of cells passed through Matrigel invasion chamber/no. of cells passed through control inserts).

### Flow cytometry and FACS

Flow cytometry and CSC gating were performed as previously described [28]. Briefly, cells were stained with anti-CD44-APC (559942; BD Biosciences, Franklin Lakes, NJ), anti-CD24-PE (555428; BD Biosciences), anti-EpCAM-PerCP-Cy5.5 (347199; BD Biosciences) antibodies, Hoechst 33258 (BD Biosciences), propidium iodide (PI; 556463; BD Biosciences), and Annexin V (Biolegend, San Diego, California; 640918). Flow cytometric analysis was performed on single cell suspensions for the CD44^hi^/CD24^lo^/EpCAM^+^ CSC phenotype.

### Immunofluorescence microscopy

Cells were permeabilised by incubating with 1% Triton X-100 for 20 min. Cells were probed with rabbit anti-DUSP1 (MKP-1; sc-370; Santa Cruz), mouse anti-DUSP4 (MKP-2; sc-17821; Santa Cruz), goat anti-DUSP6 (MKP-3; sc-8598; Santa Cruz), anti-H3K4me1 (ab8895; Abcam, Cambridge, UK), anti-H3K27ac (ab4729; Abcam), anti-H3K9me1 (S200554; Merck Millipore), anti-H3K9me3 (07–442; Merck Millipore), rabbit anti-p300 (sc-585; Santa Cruz) rabbit anti-p300-1834p (PA5-12735; Thermofisher) or rabbit anti-p300-89p (sc-130210; Santa Cruz) followed by visualisation with secondary rabbit (A10042; Life Technologies), mouse (A11001; Life Technologies), or goat (A11055; Life Technologies) immunoglobulins conjugated to Alexa Fluor 488 or 568. Cover slips were mounted on glass microscope slides with ProLong Gold Antifade^®^ reagent (Life Technologies). DUSPs were localised by confocal laser scanning microscopy. Single 0.5 μm sections were obtained using a Nikon x 60 oil immersion lens on the Nikon C1 plus confocal system running NIS-Elements AR 3.2 software. The final image was obtained by averaging four sequential images of the same section. Digital confocal images were analysed using ImageJ software (ImageJ, NIH, Bethesda, MD, USA) to determine the nuclear/cytoplasmic fluorescence ratio (Fn/c) using the equation: Fn/c = (Fn − Fb)/(Fc − Fb), where Fn is nuclear fluorescence, Fc is cytoplasmic fluorescence, and Fb is background fluorescence. Plot-profiles were plotted with the use of ImageJ software measuring a series of fluorescence intensities along a line spanning the nucleus. The pattern of the two plots provides insights into the nature of the relationship between the two fluorochromes. ImageJ software with automatic thresholding and manual selection of regions of interest (ROIs) specific for cell nuclei was used to calculate the Pearson’s co-efficient correlation (PCC) for each pair of antibodies (as described in [29,30]). PCC values range from: -1 = inverse of co-localisation, 0 = no co-localisation, +1 = perfect co-localisation. Total nuclear florescence intensity was also measured in a minimum of n = 20 cells for each sample set. Nuclear intensity was analysed using ImageJ software, with the nucleus of each cell and total nuclear fluorescence computed by the software minus background. The Mann–Whitney non-parametric test (GraphPad Prism, GraphPad Software, San Diego, CA) was used to determine significant differences between datasets.

### RNA extraction and real-time PCR analysis

Total RNA was extracted from cells using TRI Reagent^®^ (Sigma-Aldrich) as previously described [31]. 1 μg of extracted RNA was subsequently treated with recombinant DNase I (Roche Life Science, Penzberg, Germany) and reverse-transcribed using the Maxima First Strand cDNA Synthesis kit (Thermo Fisher Scientific, Waltham, MA) according to the manufacturer’s protocol. TaqMan real-time PCR was performed as previously described [31]. The human TaqMan probe sets used were: *DUSP1* (Hs00610257_g1), *DUSP4* (Hs01027785_m1), *DUSP6* (Hs04329643_s1), *FN1* (Hs00415006_m1), *VIM* (Hs00185584_m1), *SNAI1* (Hs00195591_m1), *SNAI2* (Hs00950344_m1), *CDH1* (Hs00170423_m1), *CD44* (Hs00153304_m1), *PLAUR* (Hs00182181_m1), and *PPIA* (Hs99999904_m1).

### Chromatin immunoprecipitation

Chromatin immunoprecipitation (ChIP) assays were performed as previously described [28]. Briefly, cells were fixed in 1% paraformaldehyde and sonicated with a Misonix Sonicator S-4000 (Cole-Parmer, London, UK) under conditions that produce approximately 400 bp DNA fragments. Samples were incubated overnight at 4°C with Protein A magnetic beads (Millipore, Billerica, MA) and 5 μg of anti-rabbit DUSP1 (sc-370), anti-mouse DUSP4 (sc-17821), or anti-goat DUSP6 (sc-8598) antibodies. Immune complexes were washed then eluted, and protein-DNA cross-links were reversed by incubating overnight at 66°C. DNA was extracted by phenol-chloroform extraction and precipitated by ethanol. A no antibody IP control and total input control were included in all ChIP assays to ensure specific enrichment compared to total genomic DNA.

### Immunoblot analysis

Immunoblot analysis of nuclear and cytoplasmic extracts from DUSP siRNA untreated or treated MCF-7 cells was performed using primary anti-rabbit DUSP1 (sc-370), anti-mouse DUSP4 (sc-17821), or anti-goat DUSP6 (sc-8598) antibodies and secondary HRP-conjugated anti-rabbit (AP187P; Merck Millipore), anti-mouse (AP181P; Merck Millipore), or anti-goat (sc-2020; Santa Cruz) antibodies. Signals were detected by enhanced chemiluminescence with film exposure. Bands of the correct MW were analysed with ImageJ for intensity, and protein loading was normalised with a highly sensitive, quantitative total protein loading control detection kit (Novex^®^ Reversible Membrane Protein Stain Kit).

### Half-chromatin immunoprecipitation (ChIP) assays

Half-ChIP assays were performed according to the manufacturer’s instructions (Upstate Biotechnology) and as previously described for Jurkat T cells [32]. Fixation was performed as detailed, and fixed chromatin was sonicated with an Ultrasonic processor (Qsonica) under optimised conditions to produce average DNA fragments of approximately 500 base pairs (bp). Soluble chromatin fraction was incubated overnight at 4°C with a primary antibody to DUSP4 (sc-17821) and Protein A magnetic beads or a no antibody negative control. The beads were washed and incubated with immunoblot loading buffer containing beta-mercaptoethanol at 95°C and analysed as above (immunoblot analysis) with a primary antibodies targeting p300-1834p (PA5-12735), RNA-Pol-II-serine2 (ab5095), or a RNA-Pol-II-serine5 (ab5131).

### ChIP-seq

The PKC-θ siRNA ChIP-seq data discussed in this publication were obtained through GEO Series accession number GSE53320. Library preparation and bioinformatics analysis were performed as previously described [28,33].

### Tissue samples and immunohistochemistry

Donors tissue came from patients who had consented to be part of the breast cancer treatment group database (http://www.health.act.gov.au/research-publications/research/breast-cancer-research), which includes detailed clinicopathological information including receptor status and patient follow-up. Names were extracted from this database. Sequential patients were selected based on ER/PR/HER2 status and where there was more than 10 years clinical follow up. Once the blocks were retrieved, the cases were anonymised and de-identified so the pathologist was unaware of the patient or their treatment group when reviewing the slides. The inclusion criteria were more than 10 years follow-up and a histological diagnosis of invasive ductal carcinoma. The exclusion criteria were not enough tissue in the block and male breast cancer. Consent for use of archival tissue blocks was obtained from the University of Canberra Human Ethics Committee approval number HREC 12–223.

### Statistical analysis

All comparisons between treated and untreated samples were performed using two-way analysis of variance (ANOVA). For multiple comparisons, Sidak’s multiple comparisons test was performed unless otherwise stated. Where applicable, statistical significance is denoted by * for P ≤ 0.05, ** for P ≤ 0.01, *** for P ≤ 0.001, and **** for P ≤ 0.0001. Data are expressed as mean ± standard error (SE).

## Results

### DUSPs are induced during EMT in breast cancer cells in a PKC-θ-dependent manner

**Fig 1:**
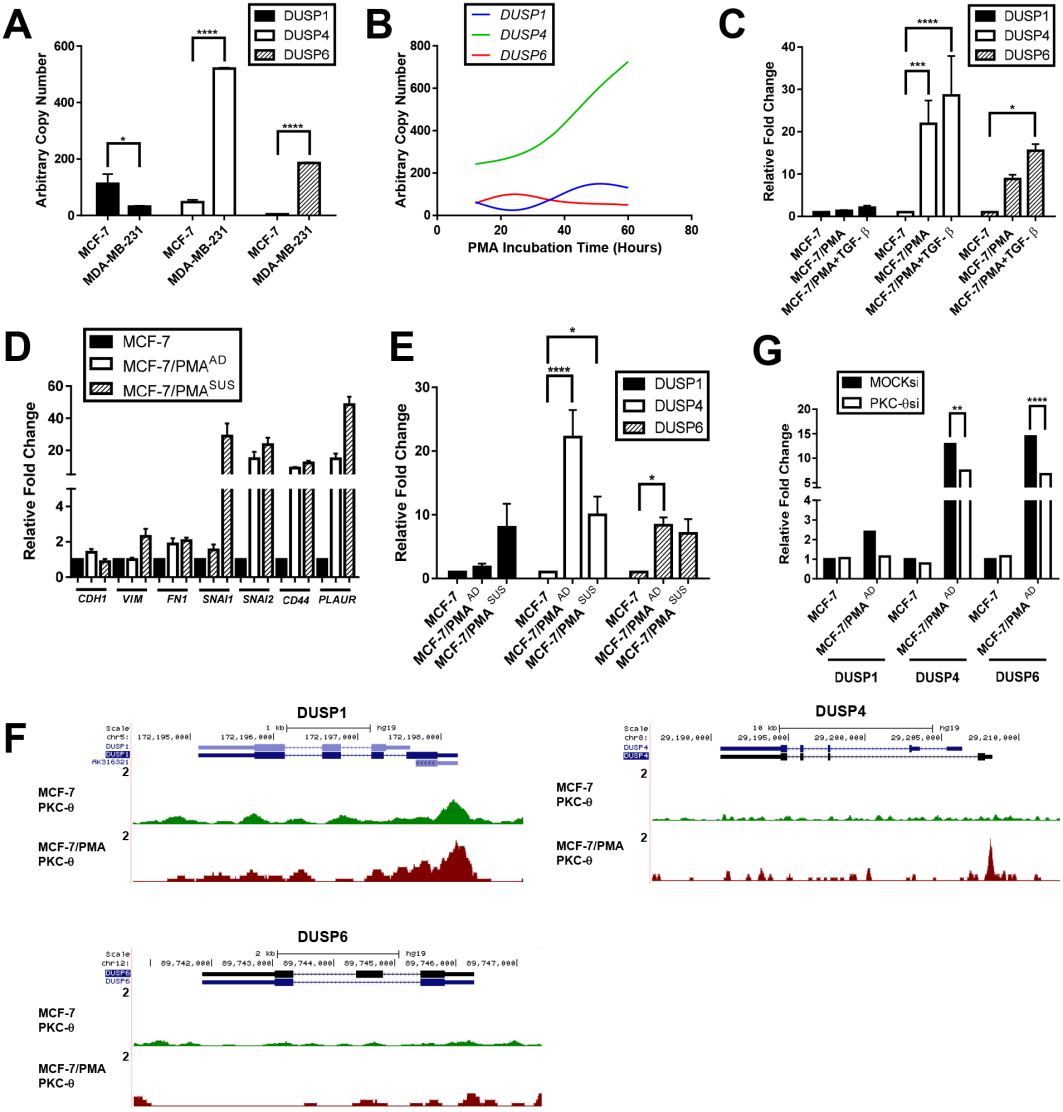
DUSPs are induced during epithelial-to-mesenchymal transition. *DUSP1*, *DUSP4*, and *DUSP6* transcript levels measured by real-time PCR in (**A**) MCF-7 and MDA-MB-231 cells. Data are expressed as arbitrary copy numbers normalised to *PPIA* and are representative of the mean ± SE. (**B**) MCF-7 cells after incubation with PMA for various periods of time. Spline curves represent mean arbitrary copy numbers normalised to *PPIA* of two independent experiments. (**C**) MCF-7 cells after incubation with vehicle alone or in the presence of either PMA or PMA+TGF-β for 60 h. Data are expressed as relative fold change to MCF-7 normalised to *PPIA* and are representative of the mean ± SE of four independent experiments. MCF-7 cells were incubated with vehicle alone or in the presence of PMA for 60 h. Transcript levels measured by real-time PCR of: (**D**) *CDH1*, *VIM*, *FN1*, *SNAI1*, *SNAI2*, *CD44*, and *PLAUR* and (**E**) *DUSP1*, *DUSP4*, and *DUSP6*. Data are presented as relative fold change to MCF-7 normalised to *PPIA* and are representative of the mean ± SE. (**F**) *DUSP1*, *DUSP4*, and *DUSP6* transcript levels in PKC-θ siRNA transfected cells. Data are expressed as relative fold change to MCF-7 MOCK siRNA normalised to *PPIA* and are representative of the mean ± SE for four experiments. (**G**) PKC-θ ChIP-seq across *DUSP1*, *DUSP4*, and *DUSP6* transcripts in MCF-7 and MCF-7/PMA cells. Data are shown in the UCSC Genome Browser. The scale in all UCSC images is indicated on the y-axis as numbers in reads per million mapped reads.

DUSP1, DUSP4, and DUSP6 were constitutively expressed in both epithelial MCF-7 and mesenchymal MDA-MB-231 breast cancer cell lines. Interestingly, DUSP1 expression was higher in MCF-7 cells, whereas DUSP4 and DUSP6 expression were higher in MDA-MB-231 cells (Fig 1A). Since many DUSP family members are known to be intermediate early response genes, we investigated their induction during EMT [34] in MCF-7 cells, which are known to undergo EMT after treatment with the EMT inducer Phorbol 12-myristate 13-acetate (PMA) [28,35]. DUSP1, DUSP4, and DUSP6 were all induced after PMA treatment with distinct expression profiles (Fig 1B). DUSP1 expression was reduced at 24 h and then increased and peaked at around 50 h, whereas DUSP4 expression was highly induced and rapidly increased and DUSP6 expression peaked at 24 h then declined. Furthermore, induction of all DUSP family members (1, 4, 6) in MCF-7 cells was potentiated following stimulation with TGF-β [10,28], the well-established EMT-inducer, in combination with PMA (Fig 1C).

Previous studies have shown that some DUSP family members are overexpressed in malignant breast tissues [14–16]. Thus, we addressed whether DUSPs were further upregulated in cells with greater metastatic potential *in vitro*. MCF-7 cells were treated with PMA for 60 h, resulting in the generation of two populations of mesenchymal cells: an adherent population and a metastatic suspended population denoted MCF-7/PMA^AD^ (previously referred to as MCF-7/PMA) and MCF-7/PMA^SUS^, respectively. MCF-7/PMA^SUS^ cells were significantly more invasive than MCF-7/PMA^AD^ and MCF-7 cells in an *in vitro* Matrigel invasion assay (Fig A in S1 Fig). Furthermore, a CD44^hi^/CD24^lo^/EpCAM^+^ breast CSC-like cell population was formed in MCF-7/PMA^AD^ cells, which increased significantly in MCF-7/PMA^SUS^ cells (Fig B in S1 Fig).

Several characteristic molecular changes occur during EMT, including loss of the epithelial protein E-cadherin and acquisition of mesenchymal proteins such as vimentin and fibronectin [3]. These changes are largely mediated by a cohort of EMT-TFs including Snail and Slug [6,7]. Therefore, we next analysed expression of several EMT and CSC genes in MCF-7/PMA^SUS^ cells and observed a slight reduction in *CDH1* mRNA and induction of *VIM*, *FN1*, *SNAI1*, *SNAI2*, *CD44*, and *PLAUR* (Fig 1D). Moreover, *DUSP1* expression was higher in MCF-7/PMA^SUS^ cells, while *DUSP4* expression was reduced in MCF-7/PMA^SUS^ compared to MCF-7/PMA^AD^ cells; *DUSP6* expression remained stable (Fig 1E).

PMA is a well-known PKC and inflammatory signal-mediated stimulus [36]. We have previously shown that the novel PKC family member PKC-θ directly regulates EMT and CSC signature genes in the context of the chromatin template in breast cancer [28]. Thus, we investigated whether PKC-θ regulates DUSP expression by PKC-θ ChIP sequencing (ChIP-seq). ChIP-seq revealed increased PKC-θ binding at the transcription start site (TSS) of the *DUSP1* and *DUSP4* core promoters, suggesting that PKC-θ directly regulates their induction after treatment with PMA. However, PKC-θ does not bind to any regions of the *DUSP6* gene (Fig 1F). Using PKC-θ siRNAs and MOCK treated MCF-7 cells left alone or stimulated with PMA, quantitative real-time PCR analysis showed that *DUSP1*, *DUSP4*, and *DUSP6* expression were dependent on PKC-θ (Fig 1G). Overall, these data suggest that DUSPs are induced during EMT in a PKC-θ-dependent manner where they have distinct expression profiles in subsets of mesenchymal breast cancer cells.

### DUSP family members co-localise with distinct histone modifications

**Fig 2:**
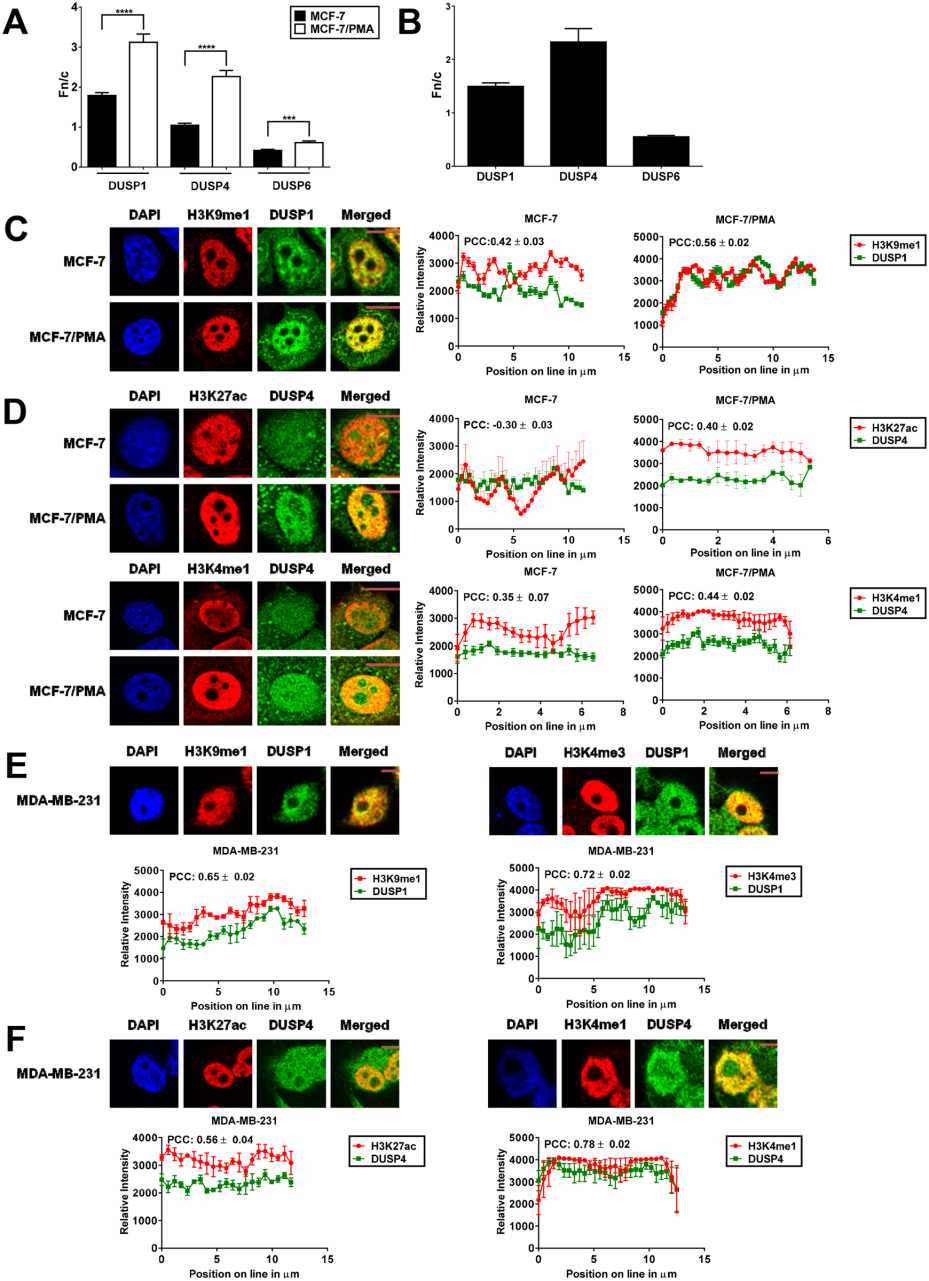
DUSPs show distinct localisation in the mesenchymal state. Confocal laser scanning microscopy was performed on either MDA-MB-231 cells or MCF-7 cells treated with vehicle alone or PMA for 60 h. Cells were fixed and probed with primary rabbit, mouse, or goat antibodies to DUSP1, DUSP4, and DUSP6, respectively, followed by the corresponding secondary antibody conjugated to Alexa-Fluor 568 or Alexa-Fluor 488. Fn/c values for: (**A**) MCF-7 and MCF-7/PMA cells; (**B**) MDA-MB-231 cells. Data shown represent the mean ± SE. Value 1 = equal cytoplasmic and nuclear fractions; >1 = nuclear bias; <1 = cytoplasmic bias. MCF-7 cells were probed with either: (**C**) primary anti-rabbit-DUSP1 and anti-mouse-H3K9me1; or (**D**) primary anti-mouse-DUSP4 and anti-rabbit-H3K27ac or anti-rabbit-H3K4me1. All antibodies were conjugated to Alexa-Fluor 568 or Alexa-Fluor 488. MDA-MB-231 cells probed with either: (**E**) primary anti-rabbit-DUSP1 and anti-mouse-H3K4me3 or anti-mouse-H3K9me1 or (**F**) primary anti-mouse-DUSP4 and anti-rabbit-H3K4me1 or anti-rabbit-H3K27ac. All antibodies were conjugated to Alexa-Fluor 568 or Alexa-Fluor 488. Representative images for each dataset are shown: green = DUSPs; red = histone marks; yellow = overlap between DUSPs and histone mark fluorescence signals. The plot-profile feature of ImageJ was used to plot the fluorescence signal intensity along a single line spanning the nucleus (n = 5 lines per nucleus, 5 individual cells). The average fluorescence signal intensity for the indicated pair of antibodies was plotted for each point on the line ±SE. Signal was plotted to compare how the signals for each antibody varied compared to the opposite antibody. The PCC was determined for each plot profile. PCC indicates the strength of relation between the two fluorochrome signals for at least 20 individual cells ± SE. Colours from representative images correspond to plot profiles.

We were interested in determining if the subcellular distribution of DUSP1, DUSP4, and DUSP6 is altered in mesenchymal cells. Typically, DUSP1 and DUSP4 localise in the nucleus, while DUSP6 localises in the cytoplasm [37]. Fluorescence microscopy revealed that DUSP1 displayed a strong nuclear bias in MCF-7 cells that increased in MCF-7/PMA cells, whilst the nuclear and cytoplasmic distribution of DUSP4 was equal in MCF-7 cells but became strongly nuclear in MCF-7/PMA cells. Conversely, DUSP6 was predominantly cytoplasmic in MCF-7 cells and, while there was significant translocation to the nucleus in MCF-7/PMA cells, remained predominantly located in the cytoplasmic compartment (Fig 2A). Furthermore, in MDA-MB-231 cells, DUSP1 and DUSP4 were predominantly localised in the nucleus while DUSP6 showed strong cytoplasmic bias (Fig 2B).

Highly compacted chromatin structures are enriched in nucleosomes and are transcriptionally silent. Chromatin remodelling is pivotal in regulating gene expression and can be orchestrated via a number of mechanisms including the addition of post-translational modifications (PTMs) to histone proteins [38]. Given the increased nuclear bias of DUSPs in MCF-7/PMA cells, we investigated co-localisation of DUSPs with several PTMs to elucidate potential roles for these family members. DUSP1 displayed clear co-localisation with H3K9me1, a mark of active promoters [39], in MCF-7 cells (PCC = 0.42), which displayed a strong increase in MCF-7/PMA cells (PCC = 0.56) (Fig 2C). However, DUSP1 co-localised with the active promoter mark [39] H3K4me3, which slightly decreased in MCF-7/PMA cells (PCC = 0.37) compared to MCF-7 cells (PCC = 0.44) (Fig A in S2 Fig). DUSP4 co-localised with the enhancer mark H3K27ac [40] in MCF-7/PMA (PCC = 0.40) but not in non-stimulated MCF-7 cells (PCC = -0.30). Moreover, DUSP4 co-localised with the enhancer mark H3K4me1 [40] in MCF-7 cells (PCC = 0.35), which increased in MCF-7/PMA cells (PCC = 0.44) (Fig 2D). In comparison, there was absent or minimal co-localisation of DUSP4 with H3K4me3, H3K4me1, and H3K9me3 in MCF-7 and MCF-7/PMA cells (Fig B in S2 Fig). Conversely, DUSP6 was expressed at very low levels or did not co-localise with any of the tested histone modifications in MCF-7 or MCF-7/PMA cells (Fig A in S3 Fig).

Similar co-localisation studies were conducted in MDA-MB-231 cells to further understand the potential contribution of DUSP family members in the mesenchymal state. Similar to above, DUSP1 showed high levels of co-localization with the active promoter marks H3K9me1 (PCC = 0.65) and H3K4me3 (PCC = 0.72) (Fig 2E), while DUSP4 showed definite co-localisation with the enhancer marks H3K27ac (PCC = 0.56) and strong co-localisation with H3K4me1 (PCC = 0.78) (Fig 2F) but not H3K4me3 or H3K9me3 (Fig C in S2 Fig). DUSP6 did not co-localise with any of the tested histone modifications in MDA-MB-231 cells (Fig B in S3 Fig). Overall, these data suggest that DUSP1, DUSP4, and DUSP6 are differentially localised in the mesenchymal state, co-localising with different histone modifications. Therefore, they are likely to play distinct roles in gene regulation.

### DUSP1 and DUSP4 have a chromatin-binding role in the mesenchymal state

**Fig 3:**
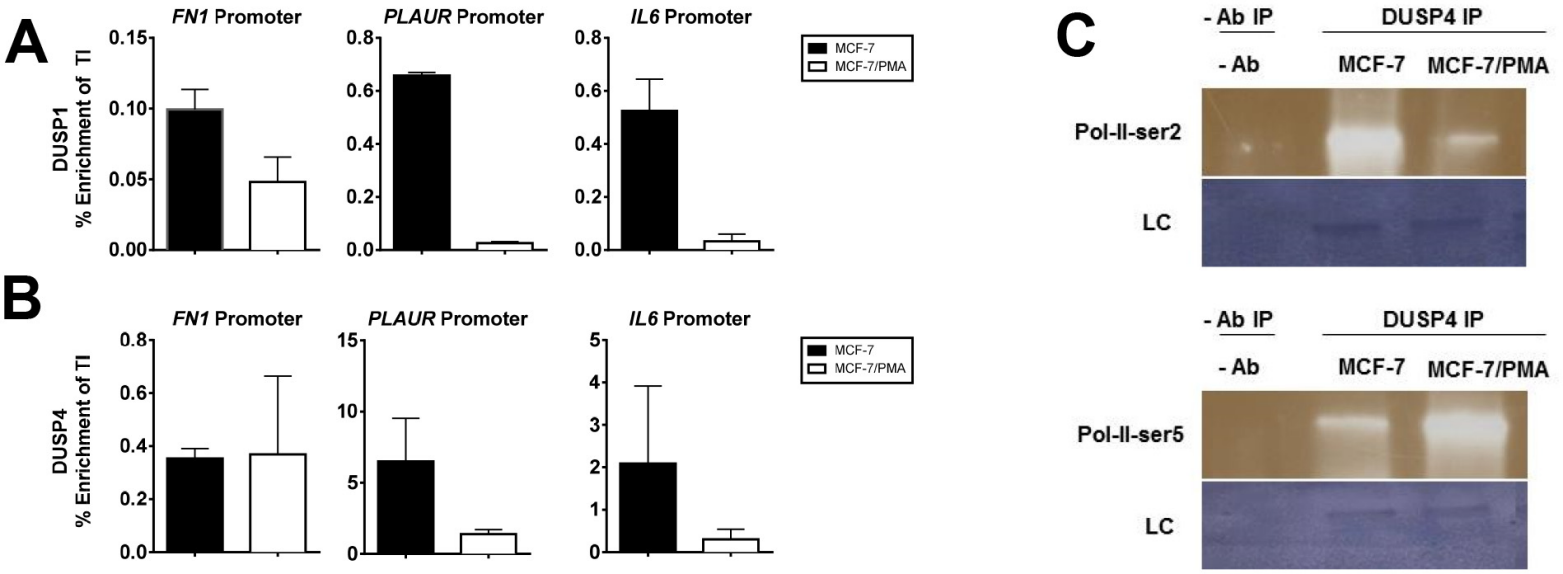
DUSP1 and DUSP4 directly tether to the promoters of mesenchymal genes. MCF-7 cells were incubated with either vehicle alone or with PMA for 60 h. ChIP was performed with DUSP1 and DUSP4 antibodies. ChIP DNA was analysed by SYBR Green real-time PCR. Enrichment across the promoter regions of *FN1*, *PLAUR*, and *IL6* are shown for: (**A**) DUSP1 and (**B**) DUSP4. Data are expressed as percentage enrichment relative to total input control and represent the mean ± SE of three independent experiments. (**C**) Nuclear extracts were obtained from MCF-7 cells stimulated as previously described and subjected to half-ChIP using DUSP4 pull down or a no antibody control. Immunoblots of the samples were probed with primary rabbit antibodies to RNA-Pol-II-serine2p or RNA-Pol-II-serine5p and detected as described in the methods. Representative images of the immunoblots are depicted along with the Novex loading control (LC).

Given the high nuclear fraction of DUSP1 and DUSP4 and their co-localisation with histone modifications, we postulated that these DUSPs directly tether to chromatin. Using ChIP, we examined DUSP1, DUSP4, and DUSP6 occupancy across the promoters of two key EMT genes, *FN1* and *PLAUR*, and the inflammatory gene *IL6*. DUSP1 was enriched at the promoter region of *FN1*, *PLAUR*, and *IL6* in MCF-7 cells, which decreased in MCF-7/PMA cells (Fig 3A). Similarly, DUSP4 was enriched at the promoters of these genes in MCF-7 cells and displayed reduced enrichment in MCF-7/PMA cells at *PLAUR* and *IL6* but not *FN1* gene promoters (Fig 3B). As expected, given the predominantly cytoplasmic localisation of DUSP6, it was not enriched at the promoters of these genes (data not shown). Furthermore, DUSP4 half-ChIP on MCF-7 and MCF-7/PMA^AD^ nuclear extracts showed that DUSP4 associates with both key phosphorylation residues (Ser2 and Ser5) of a key indicator of active chromatin, RNA Polymerase II (Fig 3C). Interestingly, we found that siRNA-mediated knockdown of DUSP1 and DUSP4 does not affect the expression of these genes (data not shown). Taken together, these data indicate a novel chromatin-anchored role for DUSP1 and DUSP4 in mesenchymal breast cancer cells.

### DUSP4 regulates p300 phosphorylation during EMT in breast cancer cells

**Fig 4:**
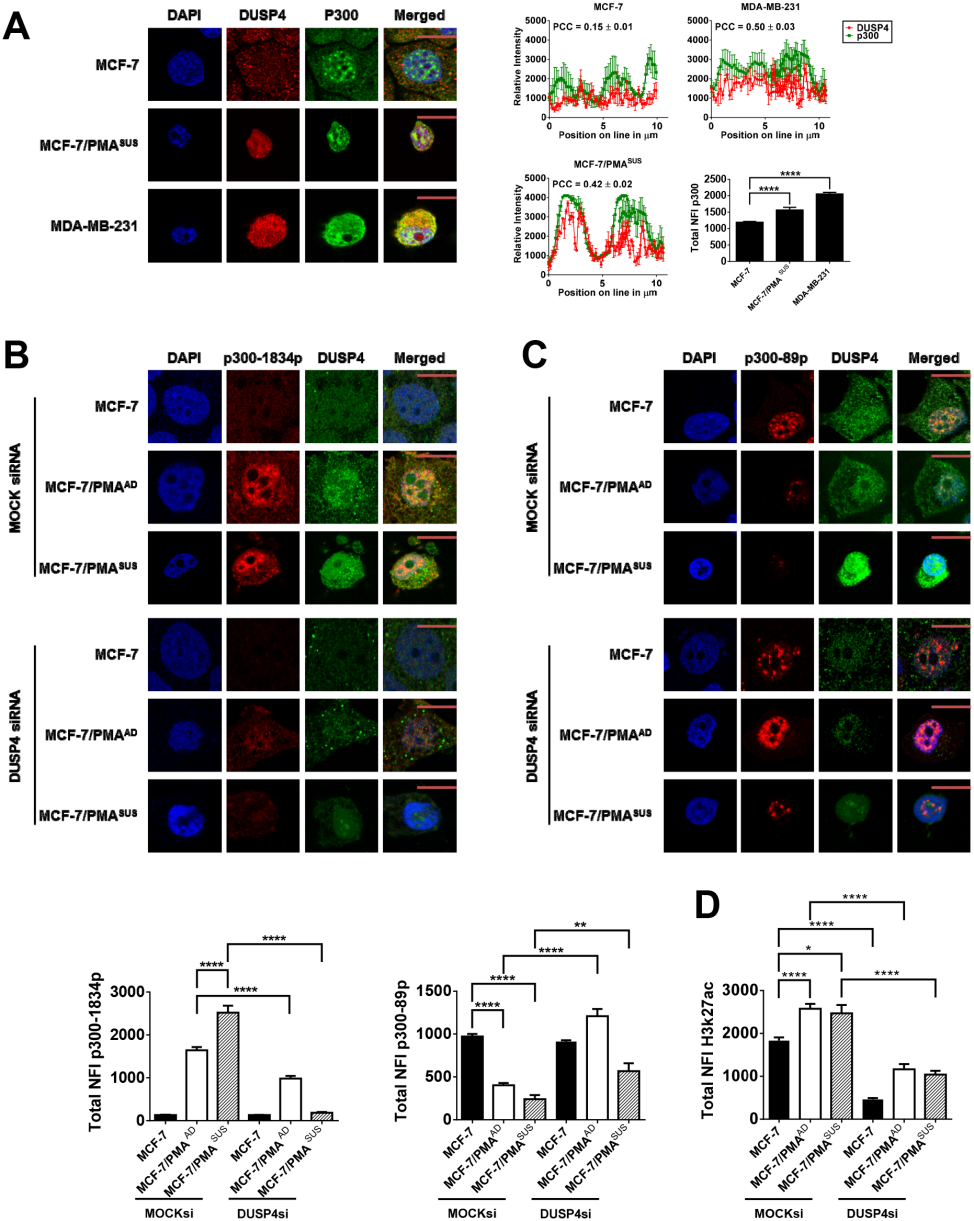
DUSP4 regulates p300 phosphorylation status. Confocal laser scanning microscopy was performed on either MDA-MB-231 cells or MCF-7 cells treated with vehicle alone or PMA for 60 h (MCF-7/PMA^AD^ or MCF-7/PMA^SUS^ cells) and subsequently transfected with either MOCK siRNA or DUSP4 siRNA. Cells were fixed and probed with primary rabbit antibodies to p300 (**A**), p300-1834p (**B**), or p300-89p (**C**) and primary mouse antibodies to DUSP4 followed by the corresponding secondary antibody conjugated to Alexa-Fluor 568 or Alexa-Fluor 488. Representative images for each antibody dataset pair are shown: green = p300; red = DUSP4; yellow = overlap between DUSP4 and p300 fluorescence signals. The total nuclear intensities of p300 (**A**), p300-1834p (**B**), p300-89p (**C**), DUSP4 (**D**) and H3K27ac were also measured using ImageJ software to select the nucleus of each cell and measure the total NFI signal minus background for at least 20 individual cells ± SE plotted in the bar graphs.

Our results thus far suggest that DUSP4 has a novel chromatin-associated role and it co-localises globally with the enhancer mark H3K27ac. Given that the key histone acetyltransferase p300 mediates acetylation of this mark [41,42], we speculated that DUSP4 may regulate p300 chromatin dynamics in the mesenchymal state in breast cancer cells. Immunofluoresence analysis showed that the nuclear fluorescence intensity of p300 increased in the mesenchymal state, in MCF-7/PMA^SUS^ and MDA-MB-231 cells compared to MCF-7 cells (Fig 4A). Moreover, DUSP4 and p300 significantly co-localise in MCF-7/PMA^SUS^ (PCC = 0.42) and MDA-MB-231 cells (PCC = 0.50) and minimally co-localised in MCF-7 cells (PCC = 0.15) (Fig 4A). Consistent with these findings, DUSP4 half-ChIP on MCF-7 and MCF-7/PMA^AD^ nuclear extracts showed that DUSP4 increased its association with p300 in the mesenchymal state (S4 Fig).

Phosphorylation of p300 at Ser-1834 is essential for its histone acetyltransferase activity [43]. In contrast, phosphorylation of p300 at Ser-89 represses its activity [44]. Next, we investigated the impact of DUSP4 knockdown on p300 phosphorylation by immunofluorescence analysis of either MOCK or validated DUSP4 siRNA-treated MCF-7 cells prior and subsequent to stimulation with PMA. DUSP4 knockdown was first confirmed by real-time PCR and immunoblotting (Figs A, B, C in S5 Fig).

In MOCK treated cells, confocal microscopy revealed increased p300-1834p nuclear fluorescence intensity in MCF-7/PMA^AD^ and MCF-7/PMA^SUS^ cells which was significantly abrogated following DUSP4 knockdown (Fig 4B). In comparison, MOCK treated samples displayed the p300 repressive phosphorylation mark, p300-89p, in MCF-7 cells and significantly lower in MCF-7/PMA^AD^ and MCF-7/PMA^SUS^ cells. However, p300-89p significantly increased after DUSP4 knockdown in MCF-7/PMA^AD^ and MCF-7/PMA^SUS^ cells (Fig 4C). Furthermore, DUSP4 knockdown inhibited H3K27ac in these cells (Fig 4D). Collectively, our findings show that DUSP4 is involved in mediating the dynamics of two key phosphorylation states, namely the active (Ser-1834) and inhibitory (Ser-89) states of p300, which are critical for the enzyme’s optimal acetyltransferase function and for acetylating the H3K27 residue.

### The PKC pathway is required for nuclear interaction of DUSP4 and p300 in EMT in breast cancer cells

**Fig 5:**
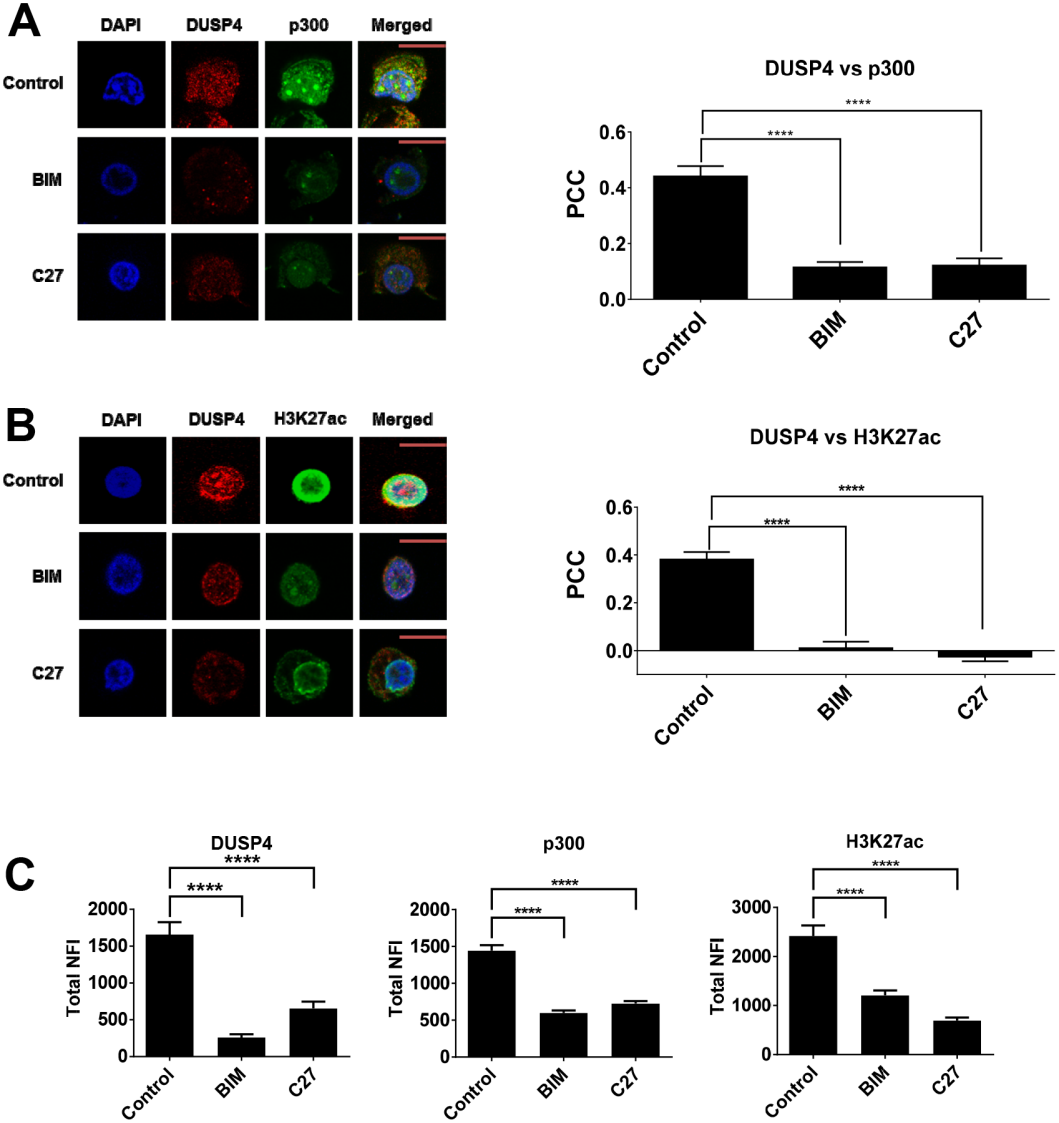
PKC-θ regulates DUSP4, p300 and H3K27ac in mesenchymal MCF-7/PMA^SUS^ cells. Confocal laser scanning microscopy was performed on MCF-7/PMA^SUS^ cells and treated with either BIM or C27 PKC-θ catalytic inhibitors. Cells were fixed and probed with primary mouse antibodies to DUSP4 and either primary rabbit antibodies to **(A)** p300 or **(B)** H3K27ac followed by the corresponding secondary antibody conjugated to Alexa-Fluor 568 or Alexa-Fluor 488. Representative images for each antibody dataset pair are shown: green = p300 or H3K27ac; red = DUSP4; yellow = overlap between DUSP4 and p300 fluorescence signals. The PCC was determined as described in methods for both MOCK control and cells treated with either BIM or C27 PKC-θ catalytic inhibitors. PCC indicates the strength of relation between the two fluorochrome signals for at least 20 individual cells ± SE. **(C)**. Bar graphs indicate the total NFI of DUSP4, p300, or H3K27ac as measured using ImageJ software to select the nucleus of each cell and measure the total NFI signal minus background for at least 20 individual cells ± SE.

Our results above suggest that the PKC pathway mediates increased coupling of DUSP4 with the key chromatin associated enzyme p300 in the mesenchymal state. To address whether this pathway is essential for this event in breast cancer cells, we assessed the impact of PKC catalytic activity using two ATP-competitive inhibitors: BIM (pan-PKC inhibitor) and C27 (PKC-θ-specific inhibitor), which we have previously shown to inhibit EMT and CSC formation in breast cancer [28]. Pre-treatment with either C27 or BIM resulted in significant reduction in the co-localisation of DUSP4 and p300 in MCF-7/PMA^SUS^ cells (PCC = 0.11 for BIM-treated cells and PCC = 0.12 for C27-treated cells) compared to vehicle treated cells (PCC = 0.42 for p300) (Fig 5A). Similarly, co-localisation of DUSP4 and H3K27ac was abrogated (PCC = 0.01 for BIM-treated cells and PCC = -0.025 for C27-treated cells) (Fig 5B). The total nuclear fluorescence signal of DUSP4, p300 and H3K27ac were also reduced (Fig 5C). Collectively, these data suggest that the PKC pathway, and in particular PKC-θ, is required for the interplay between DUSP4 and p300 and the key enhancer mark H3K27ac.

### DUSPs have variable roles in breast CSC regulation

**Fig 6:**
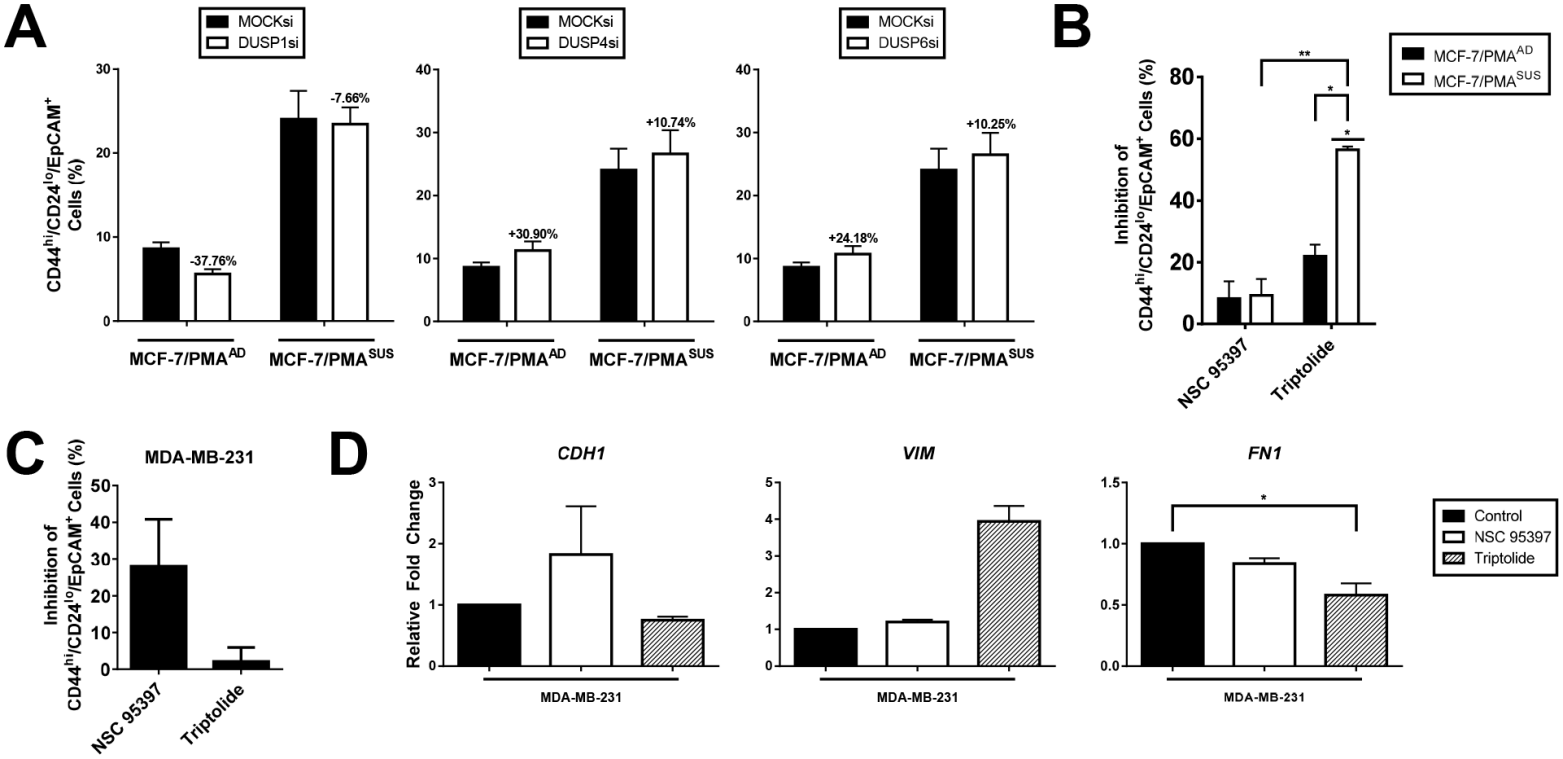
DUSPs are involved in breast CSC regulation. MCF-7 cells were transfected with MOCK, DUSP1, DUSP4, or DUSP6 siRNAs and subsequently incubated with either vehicle or in the presence of PMA for 60 h. (**A**) Percentage of CD44^hi^/CD24^lo^/EpCAM^+^ CSC-like cells as measured by flow cytometry after transfection ± SE. Numbers above each column represent percentage inhibition (-) or promotion (+) relative to MOCK siRNA for three independent experiments. MCF-7 and MDA-MB-231 were inhibited with either NSC 95397 or triptolide for 24 h. (**B**) MCF-7 cells were then incubated with either vehicle alone or PMA for 60 h. Bar graph indicates percentage inhibition of CD44^hi^/CD24^lo^/EpCAM^+^ CSC-like MCF-7, MCF-7/PMA^AD^, and MCF-7/PMA^SUS^ cells after inhibition ± SE for two independent experiments. (**C**) Bar graph indicates CD44^hi^/CD24^lo^/EpCAM^+^ CSC-like MDA-MB-231 cells after inhibition ± SE for two independent experiments. (**D**) mRNA levels *CDH1*, *VIM*, and *FN1* in MDA-MB-231 cells after inhibition. Data represent the mean relative fold change to untreated cells normalised to *PPIA* ± SE for two independent experiments.

Since DUSP1, DUSP4, and DUSP6 are induced during EMT, we utilised a siRNA-based approach to determine whether they are also involved in breast CSC formation. DUSP knockdown was first confirmed by real-time PCR and immunoblotting (Figs A, B in S5 Fig). Consistent with previous reports implicating DUSP1 in breast CSC survival [17], DUSP1 knockdown inhibited CSC formation in MCF-7/PMA^AD^ and MCF-7/PMA^SUS^ cells. Conversely, DUSP4 and DUSP6 knockdown enhanced breast CSC formation in both subpopulations (Fig 6A).

Since small molecule inhibitors are increasingly used as adjuvant anti-cancer therapies, we were interested in establishing whether DUSP inhibitors could further abrogate the formation and maintenance of breast CSCs. There is no commercially available DUSP4-specific inhibitor [45]. Thus, two non-specific small molecule inhibitors NSC 95397 (which inhibits DUSP1 and DUSP6) and triptolide (which inhibits DUSP1) were used [46–48]. Treatment with NSC 95397 inhibited CSC formation by 8–9% in both MCF-7/PMA^AD^ MCF-7/PMA^SUS^ cells, while triptolide inhibited CSC formation by 22% in MCF-7/PMA^AD^ cells and 56% in MCF-7/PMA^SUS^ cells (Fig 6B). In MDA-MB-231 cells, treatment with NSC 95397 reduced breast CSCs by 28% whilst triptolide had minimal effect (Fig 6C).

We next addressed whether these DUSPs regulate EMT-associated gene expression. Inhibition of DUSPs had differential effects in MDA-MB-231 cells: DUSP1 and DUSP6 inhibition with NSC 95397 slightly increased *CDH1* expression but had little effect on *VIM* or *FN1* expression, whilst DUSP1 inhibition alone with triptolide increased *VIM* expression, reduced *FN1* expression, and had no effect on *CDH1* expression (Fig 6D). In contrast, these data suggest that DUSPs are involved in breast CSC regulation and have varying roles in regulating EMT-associated genes.

### DUSP6 is a potential biomarker in malignant HER2+ breast cancers

**Fig 7:**
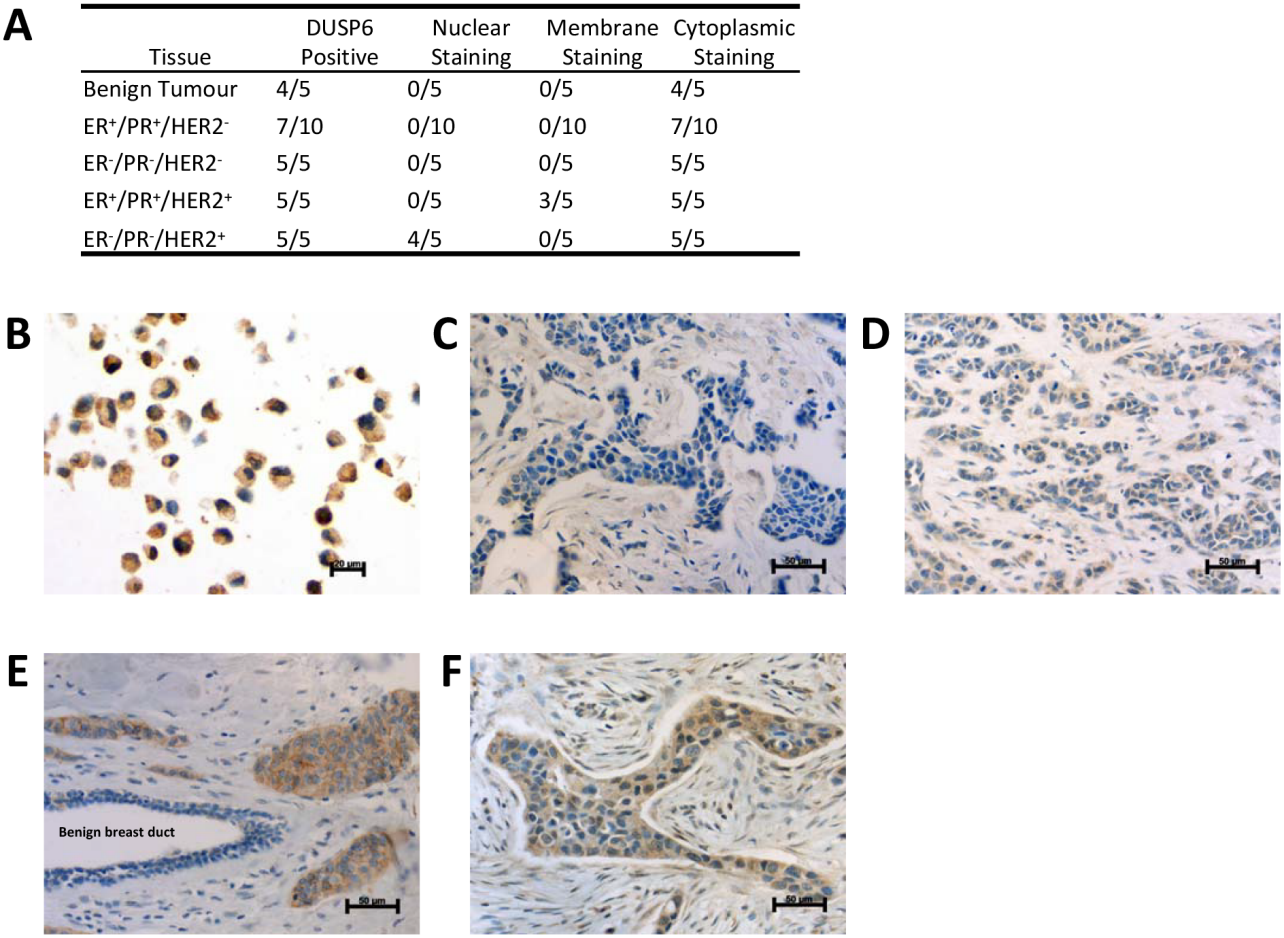
DUSP6 is moderately expressed in malignant HER2^+^ breast cancers. DUSP6 expression in a breast cancer cell line and breast cancer tissues were examined by immunohistochemistry. (**A**) Table summarising DUSP6 expression in the indicated breast tissues. (**B**) Cell block of MDA-MB-231 cells (x630). (**C**) Grade 2 ER^+^/PR^+^/HER2^-^ breast cancer (x400). (**D**) Grade 3 triple-negative (ER^-^/PR^-^/HER2^-^) breast cancer (x400). (**E**) Grade 3 ER^+^/PR^+^/HER2^+^ breast cancer (x400) with benign breast duct. (**F**) Grade 3 ER^-^/PR^-^/HER2^+^ breast cancer (x400).

Little is known about DUSP6 expression in breast cancer tissue, except that DUSP6 is upregulated in HER2^+^ breast carcinomas [21,22]. However, DUSP6 expression in benign breast tissue compared to invasive breast carcinomas has yet to be investigated. DUSP6 IHC analyses were conducted in benign and a number of malignant breast cancer tissues (Fig 7A). IHC analysis of MDA-MB-231 cells revealed moderate cytoplasmic DUSP6 expression with patchy weak nuclear staining (Fig 7B). DUSP6 expression was weak and cytoplasmic in 4/5 samples of normal breast tissue from reduction mammoplasty samples resected from patients with no known family history of breast cancer (Fig 7A). Similarly, ER^+^/PR^+^/HER2^-^ breast cancers showed weak or absent cytoplasmic DUSP6 expression (3/10) (Fig 7C), and all (5/5) triple-negative (ER^-^/PR^-^/HER2^-^) breast cancers showed weak cytoplasmic DUSP6 expression (Fig 7D). Interestingly, triple-positive breast cancers (ER^+^/PR^+^/HER2^+^) showed weak to moderate cytoplasmic DUSP6 staining (5/5) (Fig 7E). Note the weak patchy cytoplasmic DUSP6 staining in the benign breast duct (Fig 7E). Furthermore, ER^-^/PR^-^/HER2^+^ breast cancers showed weak to moderate cytoplasmic DUSP6 expression, with up to 10% of cells showing weak nuclear staining (Fig 7F). Taken together, these data suggest that DUSP6 is upregulated in malignant HER2^+^ breast cancers, regardless of ER/PR status.

## Discussion

EMT is a key step in cancer progression and enriches for a population of CSCs; they are, therefore, critical mediators of breast cancer tumourigenesis. CSCs were first implicated in breast cancer progression in 2003 [11]. Since then, they have emerged as important drivers of metastasis, resistance to conventional cytotoxic therapies, and subsequent cancer recurrence. There is currently an unmet need for the development of clinically effective drugs that target CSCs via the oncogenes and tumour suppressors that regulate their formation and maintenance.

In this study, we explored the role of three DUSP family members in EMT and the regulation of breast CSCs: DUSP1, DUSP4, and DUSP6. Specifically, we have shown that: (1) DUSP family members are induced during EMT via PKC-mediated signals; (2) nuclear crosstalk exists between the chromatin-associated kinase PKC-θ and these DUSPs, with PKC-θ directly binding to their proximal regulatory elements to regulate these phosphatases; (3) DUSP family members globally tether to distinct epigenomic compartments with specific roles in gene regulation in the mesenchymal state; (4) DUSP4 and p300 associate in the context of the chromatin template and DUSP4 regulates two key phosphorylation residues of p300 that are critical for acetylation of the H3K27 residue; and (5) DUSPs are involved in breast CSC regulation. Overall, our findings show for the first time that epigenomic crosstalk occurs between a critical chromatin-associated kinase and DUSP family phosphatases during EMT.

Our data demonstrate that DUSP1, DUSP4, and DUSP6 are induced during EMT. Their highly inducible nature is highlighted when EMT is stimulated using a combination of two potent EMT-inducers, PMA and TGF-β. Each DUSP has a distinct spatial and temporal pattern of expression during EMT, suggesting that they have specific cellular roles. Our results also show that, while DUSP1 is highly induced in metastatic MCF-7/PMA^SUS^ cells, its expression is constitutively lower in mesenchymal MDA-MB-231 cells. Conversely, the expression of DUSP4 and DUSP6, which are highly expressed in MDA-MB-231 cells, is not potentiated in MCF-7/PMA^SUS^ cells. We postulate that these DUSPs have breast cancer cell-specific effects. Indeed, although each DUSP is induced during EMT, it seems that DUSP1 may be more involved in mesenchymal transition rather than maintaining the mesenchymal state when fully differentiated. Conversely, DUSP4 and DUSP6 appear to be more involved in maintaining the mesenchymal state.

Protein kinase C (PKC) family members are pivotal proteins in inflammatory signalling and inflammation-related processes. Here we show that the PKC family member PKC-θ regulates DUSP1, DUSP4, and DUSP6 expression in response to inflammatory signals introduced via PMA and the subsequent induction of EMT. Our previous studies have shown that PKC-θ can translocate to the nucleus, anchor to chromatin, and directly regulate key EMT/CSC marker genes in breast cancer [28,32]. In this study, we have shown that PKC-θ binds to the proximal promoter regions that house the TSSs of DUSP1 and DUSP4 but not DUSP6. These interactions illustrate an interesting dynamic in which kinases selectively interact with their opposing phosphatases. It is plausible that, as well as regulating DUSP family member expression, PKC-θ may directly phosphorylate them and modulate their activity. Indeed, PKC CK2α has been shown to selectively phosphorylate DUSP6 and increase its dephosphatase activity [49]. Moreover, extracellular signal-regulated kinase 1 and 2 (ERK1/2) can phosphorylate DUSP6 to promote proteosomal degradation [50], highlighting additional layers of interaction between kinases and phosphatases.

**Fig 8:**
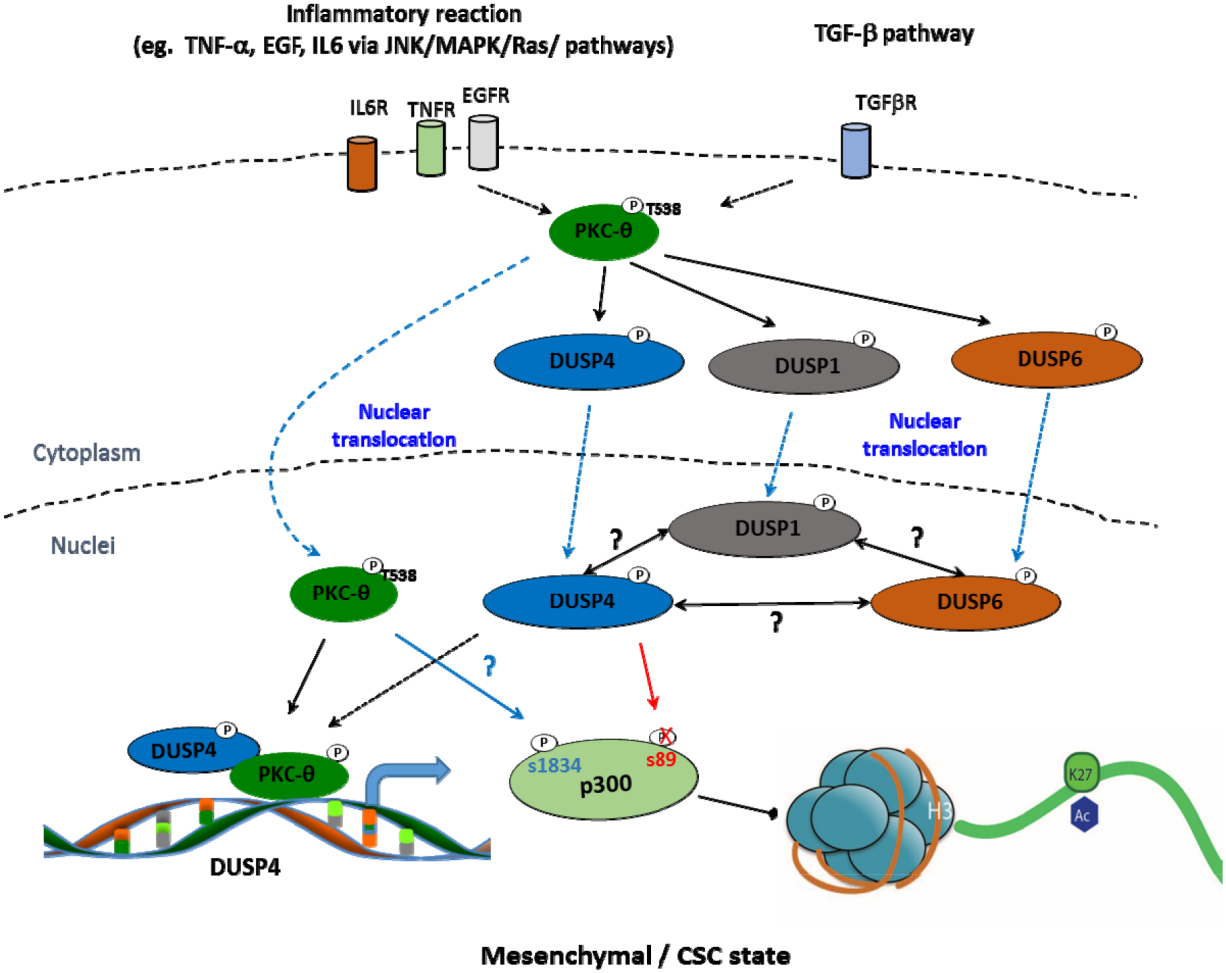
A novel role for nuclear DUSP4 in the regulation of the key epigenetic protein p300 in the mesenchymal/CSC state in breast cancer. Step 1: Activation of the PKC-θ pathway by inflammatory signals leads to DUSP4 phosphorylation and nuclear translocation. Phosphorylation of these proteins, either directly or indirectly by PKC-θ, maintains them in the nucleus [52]. PKC-θ also directly binds DUSP4 and leads to DUSP4 transcription. Step 2: DUSP4 and p300 co-exist in the epigenomic context. Step 3: DUSP4 dephosphorylates the inhibitory serine-89 phosphorylation mark of p300. Step 4: This allows phosphorylation of p300-1834 by protein kinases, such as PKC-θ and Akt [43,44]. Step 5: p300-1834p promotes H3K27 acetylation.

Our microscopy studies revealed a nuclear bias for DUSP1 and DUSP4. Furthermore, our findings at the epigenomic level suggest that DUSP family members exist within distinct epigenomic regions, decorated by distinct histone PTMs in the mesenchymal state. This suggests that each DUSP family member is likely to function on a distinct cohort of genes during EMT/CSC formation. DUSP1 is spatially organised with H3K4me3 and H3K9me1 histone modifications, both of which are associated with active promoter regions [39], while DUSP4 co-localises with H3K27ac and H3K4me1, which are marks of regulatory enhancer elements [40]. DUSP1 has previously been implicated in H3S10p dephosphorylation [51]; however, to our knowledge, DUSP4 has not been implicated as a chromatin-tethered enzyme. Our findings provide novel molecular insights into the potential nuclear functions of DUSP family members during EMT in the breast cancer setting. We show that DUSP4 co-exists with p300, a transcriptional co-activator, whereby its acetyltransferase activity mediates H3K27ac [41]. Ser-1834p is critical for the transactivation of p300 by stimulating its HAT activity, whilst Ser-89p represses the activity of p300 [43,44]. Here we show that p300-1834p is induced and p300-89p is diminished in the mesenchymal state, with maximal expression detected in the epithelial state. Intriguingly, DUSP4 knockdown resulted in inhibition of the active phosphorylation mark p300-1834p in our breast cancer cells, whilst protecting the repressive mark p300-89p. Hence, based on our findings, we propose a new model illustrating how chromatinised DUSP4 dephosphorylates p300 at serine-89, which maintains a favourable chromatin environment for 1834p (model; Fig 8) to mediate the key histone mark H3K27ac in the mesenchymal state. This work, therefore, establishes a novel role for this phosphatase in chromatin regulatory biology.

Interestingly, our ChIP analysis shows that DUSP1 and DUSP4 directly tether to key EMT/CSC inducible gene promoters. Our data suggest that tethering of DUSP1 or DUSP4 at the chromatin platform at these loci decreases in the mesenchymal state across CSC-inducible gene promoters. Furthermore, these phosphatases appear to have a minimal effect on the gene expression signature of inducible EMT genes. However, since there are 25 DUSP family members, many of which share similar domain structures, we speculate there is redundancy in their binding. This would explain why siRNA-mediated knockdown of DUSP1 and DUSP4 did not clearly affect the expression of the genes to which they tether. Additionally, we acknowledge that, although these data show that DUSP4 is enriched at promoter regions, it is possible that DUSP4 is bound to the enhancers of these genes. Due to 3D conformational interactions of chromatin and the nature of ChIP, DUSP4 may also be pulled down at physically interacting promoter regions. Further work is required to determine if such a scenario occurs and DUSP4 binds to mesenchymal gene enhancers.

Future RNA-seq of DUSP-knockdown cells will unravel how critical these DUSPs are for EMT gene expression. One possible role for DUSP1 and DUSP4 at PKC-θ-dependent gene promoters is that they mark or tag PKC-θ-responsive genes and maintain dephosphorylated histones at such loci in the epithelial state. Given that DUSP1 and DUSP4 are globally induced in response to PKC signals and exist in proximity to active proximal and distal enhancer marks in the mesenchymal state, we envisage that DUSPs may play a role in active transcription complexes and locally dephosphorylate chromatin proteins at these loci. Future ChIP-seq and sequential ChIP studies will ultimately be required to unravel the global contribution of DUSP1 and DUSP4 to breast EMT/CSCs.

An interesting finding of our work is that both positive and negative regulators of CSCs are induced during EMT. DUSP1 knockdown promoted breast CSC formation. This is consistent with a recent study implicating DUSP1 as a regulator of breast CSC survival [17]. Conversely, DUSP4 or DUSP6 knockdown repressed breast CSC formation. The involvement of all three DUSPs highlights that there is a constant balancing act between positive and negative signals in CSCs. We note that the data generated using DUSP inhibitors was inconsistent with that of the siRNA data. Treatment with triptolide (a non-specific DUSP1 inhibitor) had no effect on the constitutive CSC population in MDA-MB-231 cells; however, it did inhibit the formation of breast CSCs in MCF-7/PMA cells. Treatment with NSC 95397 (a non-specific DUSP1 and DUSP6 inhibitor) inhibited both the maintained MDA-MB-231 breast CSCs and formation of MCF-7/PMA breast CSC subpopulations. It is likely that these differential effects are due to inherent differences in the cells lines or non-specificity of the inhibitors. The development of potent, specific DUSP inhibitors is necessary to advance therapeutic targeting in the clinical setting.

The specific mechanisms by which DUSPs mediate their actions during EMT and in CSCs remain elusive. However, we speculate that it may be either entirety or partly due to the inactivation of MAPKs. Specifically, DUSP family members selectively dephosphorylate the threonine/serine and tyrosine residues of the MAPKs JNK, p38, and ERK1/2 with variable affinity [53]. JNK and p38 are stress-activated protein kinases that mediate pro-apoptotic signalling pathways [54,55]. We hypothesise that DUSP1 represses EMT and CSC regulation by dephosphorylating JNK and p38, for which it has the highest affinity [53], catalysing their inhibition and leading to JNK- and p38-induced apoptosis. PKC-θ has also been implicated in JNK activation in Jurkat T cells [56–58]. It is plausible that PKC-θ-mediated regulation of DUSP1 counteracts this activation to produce a dynamic relationship between protein kinases and phosphatases. Conversely, ERK1/2 MAPKs are well-characterised participants in cancer cell proliferation, metastasis, and EMT [59–61]. DUSP4 and DUSP6 have high affinity for ERK1/2 [53], and DUSP4 and DUSP6-mediated dephosphorylation of ERK1/2 may reduce their activities to contribute to CSC suppression.

Our microscopy and immunohistochemistry analyses reveal a strong cytoplasmic bias for DUSP6 during EMT and in both normal and malignant breast cancer tissues. Consistent with earlier reports, we found that DUSP6 is upregulated in HER2^+^ breast carcinomas [21,22] in comparison to normal mammary tissue. Interestingly, DUSP6 expression appears to be independent of ER/PR status. Given that DUSP6 overexpression in breast cancer has been linked to resistance to tamoxifen therapy [27], we postulate that HER2-mediated resistance to hormone therapies may be in part due to increased DUSP6 expression. Thus, future work should determine whether therapeutic targeting of DUSP6 potentiates responses to hormone therapies.

We must highlight that DUSP1, DUSP4, and DUSP6 have pleiotropic roles as both oncogenes and tumour suppressors in various cancers [45]. Future drug development to target these proteins must consider these variable effects. Nevertheless, we show that CSC regulation is a dynamic process between positive and negative signals, kinases, and phosphatases. We have demonstrated that DUSP1 is a promoter of breast CSCs, while DUSP4 and DUSP6 are repressors of breast CSCs. Future studies should establish the mechanisms by which DUSPs regulate breast CSCs and how they alter cellular phenotype, particularly via interactions between DUSPs and MAPKs and the effects of chromatin-anchored DUSP1 and DUSP4 in breast CSCs.

## Supporting Information

S1 Fig MCF-7 cells were incubated with vehicle alone or PMA for 60 h. (**A**) Percentage invasion through Matrigel invasion chamber. Data represent the mean ± SE of three experiments. (**B**) Flow cytometry plots with gating of CD44^hi^/CD24^lo^/EpCAM^+^ CSC-like cells and corresponding graph displaying mean percentage of CD44^hi^/CD24^lo^/EpCAM^+^ CSC-like cells ± SE from four independent experiments. (**C**) Cells were stained with Annexin V and PI and subjected to flow cytometry. The upper box indicates gating for early apoptotic cells and the lower box indicates gating for late apoptotic cells. Numbers indicate the percentage of cells in the respective gates.
(TIF)

S2 Fig Confocal laser scanning microscopy was performed on either MDA-MB-231 or MCF-7 cells treated with vehicle alone or PMA for 60 h. Cells were fixed and probed with primary rabbit or mouse antibodies to DUSP1 and DUSP4, respectively, followed by the corresponding secondary antibody conjugated to Alexa-Fluor 568 or Alexa-Fluor 488. (**A**) MCF-7 cells probed with primary anti-rabbit DUSP1 and anti-mouse H3K4me3 antibodies. (**B**) MCF-7 cells probed with primary anti-mouse DUSP4 and anti-rabbit H3K4me3, anti-rabbit H3K4me1 or anti-rabbit H3K9me3 antibodies. Representative images for above datasets are shown: green = DUSPs; red = histone marks; yellow = overlap between DUSPs and histone mark fluorescence signals. (**C**) MDA-MB-231 cells probed with primary anti-mouse DUSP4 and either anti-rabbit H3K4me3 or anti-rabbit H3K9me3 antibodies. Representative images for above dataset are shown: red = DUSPs; green = histone marks; yellow = overlap between DUSPs and histone mark fluorescence signals.
(TIF)

S3 Fig Confocal laser scanning microscopy was performed on either MDA-MB-231 cells or MCF-7 cells treated with vehicle alone or PMA for 60 h. Cells were fixed and probed with primary anti-goat DUSP6 followed by anti-goat secondary antibody conjugated to Alexa-Fluor 488. (**A**) MCF-7 cells probed with primary anti-goat DUSP6 and either anti-rabbit H3K4me1, anti-rabbit H3K4me3, anti-rabbit H3K9me3, or anti-rabbit H3K27ac antibodies. Representative images for above dataset are shown: green = DUSPs; red = histone marks; yellow = overlap between DUSPs and histone mark fluorescence signals. (**B**) MDA-MB-231 cells were probed with primary anti-goat DUSP6 and either anti-rabbit H3K4me1 or anti-rabbit H3K27ac antibodies. Representative images for above dataset are shown: red = DUSPs; green = histone marks; yellow = overlap between DUSPs and histone mark fluorescence signals.
(TIF)

S4 Fig Nuclear extracts were obtained from MCF-7 cells treated with vehicle alone or PMA for 60 h and half-ChIP using DUSP4 pull down or a no antibody control. Immunoblots of the samples were probed with either a primary rabbit antibody to a phospho-epitope of p300 (p300-1834). Band intensity was plotted using ImageJ software minus background for n = 4 with mean ± SE and normalized to the LC. Representative images of the immunoblots are depicted with along with the LC.
(TIF)

S5 Fig (**A**) Knockdown of *DUSP1*, *DUSP4*, and *DUSP6* after DUSP1 (left), DUSP4 (middle), and DUSP6 (right) siRNA transfections, respectively. Transcript levels were measured by real-time PCR and data represent the mean ± SE of five independent experiments. (**B**) Immunoblots of MCF-7 nuclear extracts (NE) and cytoplasmic extracts (CE). Extracts were probed with primary antibodies to DUSP1, DUSP4 or DUSP6, followed by the respective HRP-conjugated secondary antibody. Signals were detected with enhanced chemiluminescence reagents, bands of the correct MW were analysed with ImageJ for intensity, and the protein loading was normalised with a total protein loading control detection kit. Graph displays the relative protein band signal intensity. (**C**) Confocal laser scanning microscopy was performed on MCF-7 cells were transfected with DUSP4 siRNA and subsequently incubated with either vehicle alone or PMA for 60 h. Cells were fixed and probed with primary anti-mouse DUSP4. The total nuclear intensity of DUSP4 was measured using ImageJ software to select the nucleus of each cell and measure the total NFI signal minus background for at least 20 individual cells ± SE plotted in the bar graph.
(TIF)
