# Supplementary figures and images for: Differential Roles for DUSP Family Members in Epithelial-to-Mesenchymal Transition and Cancer Stem Cell Regulation in Breast Cancer
Source: PLoS One. 2016 Feb 9;11(2):e0148065. doi: 10.1371/journal.pone.0148065 (PMC4747493; doi:10.1371/journal.pone.0148065)

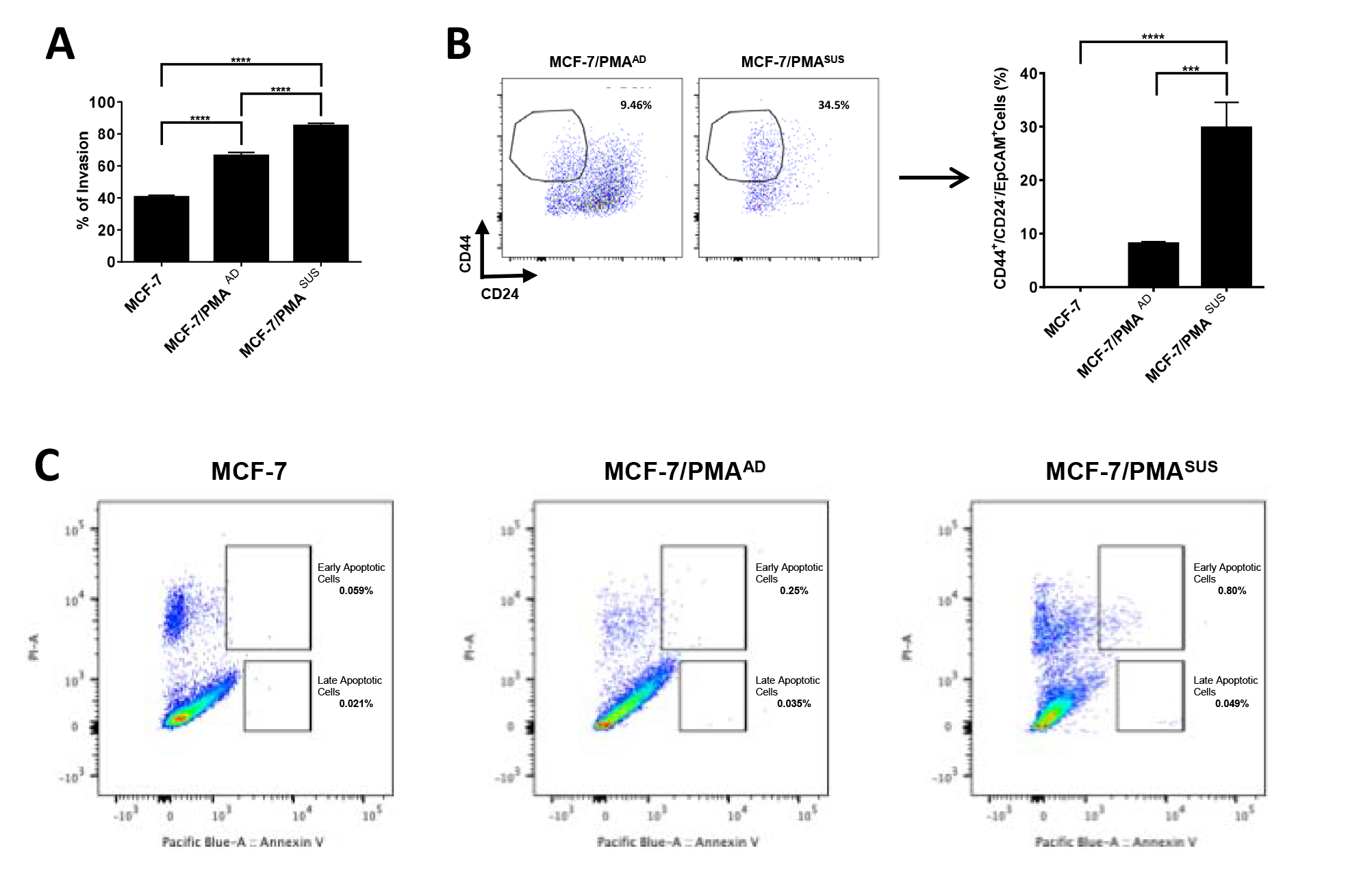

Supplement: S1 Fig — (A) Percentage invasion through Matrigel invasion chamber. Data represent the mean ± SE of three experiments. (B) Flow cytometry plots with gating of CD44hi/CD24lo/EpCAM+ CSC-like cells and corresponding graph displaying mean percentage of CD44hi/CD24lo/EpCAM+ CSC-like cells ± SE from four independent experiments. (C) Cells were stained with Annexin V and PI and subjected to flow cytometry. The upper box indicates gating for early apoptotic cells and the lower box indicates gating for late apoptotic cells. Numbers indicate the percentage of cells in the respective gates. (TIF) [file pone.0148065.s001.tif]

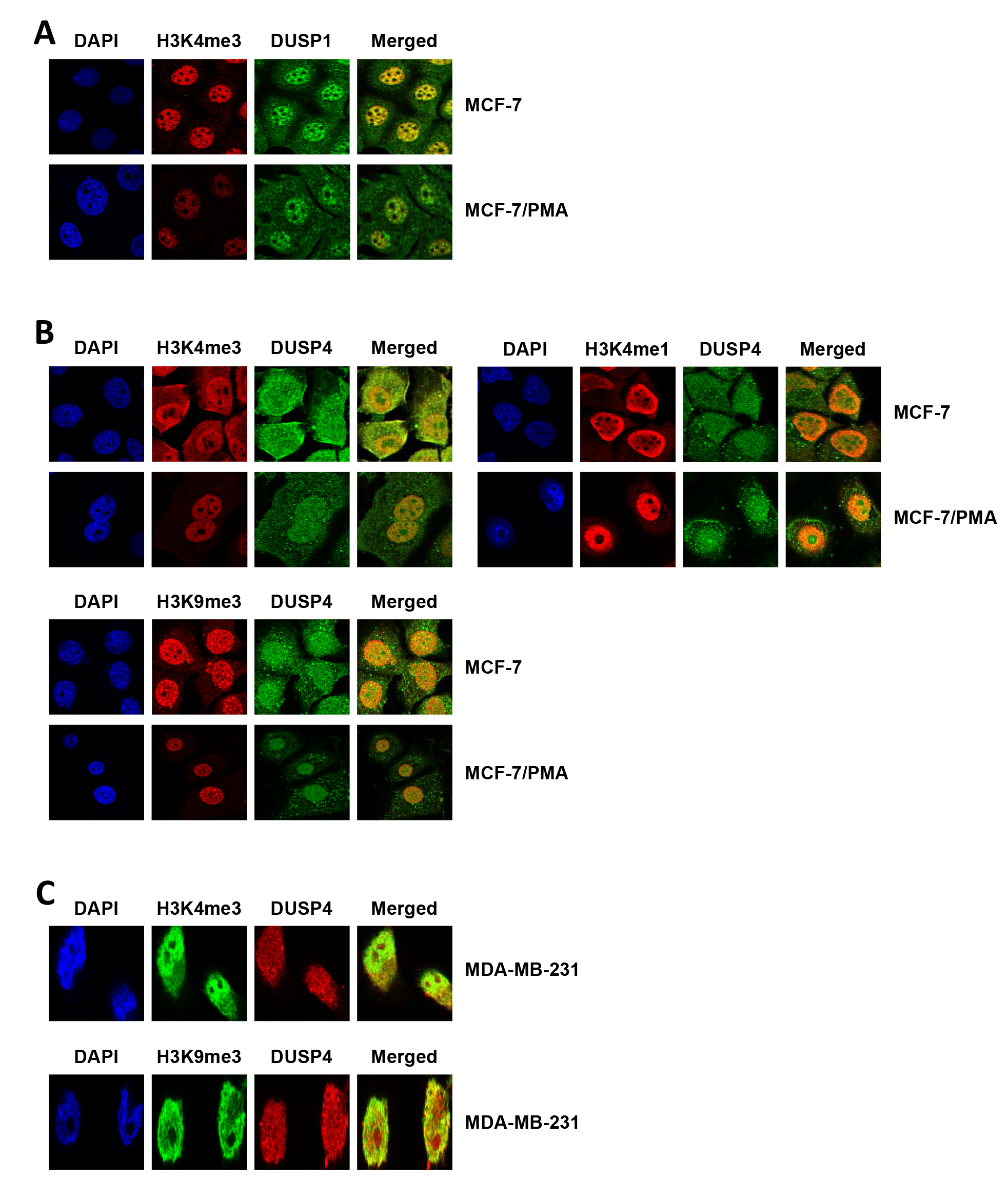

Supplement: S2 Fig — Cells were fixed and probed with primary rabbit or mouse antibodies to DUSP1 and DUSP4, respectively, followed by the corresponding secondary antibody conjugated to Alexa-Fluor 568 or Alexa-Fluor 488. (A) MCF-7 cells probed with primary anti-rabbit DUSP1 and anti-mouse H3K4me3 antibodies. (B) MCF-7 cells probed with primary anti-mouse DUSP4 and anti-rabbit H3K4me3, anti-rabbit H3K4me1 or anti-rabbit H3K9me3 antibodies. Representative images for above datasets are shown: green = DUSPs; red = histone marks; yellow = overlap between DUSPs and histone mark fluorescence signals. (C) MDA-MB-231 cells probed with primary anti-mouse DUSP4 and either anti-rabbit H3K4me3 or anti-rabbit H3K9me3 antibodies. Representative images for above dataset are shown: red = DUSPs; green = histone marks; yellow = overlap between DUSPs and histone mark fluorescence signals. (TIF) [file pone.0148065.s002.tif]

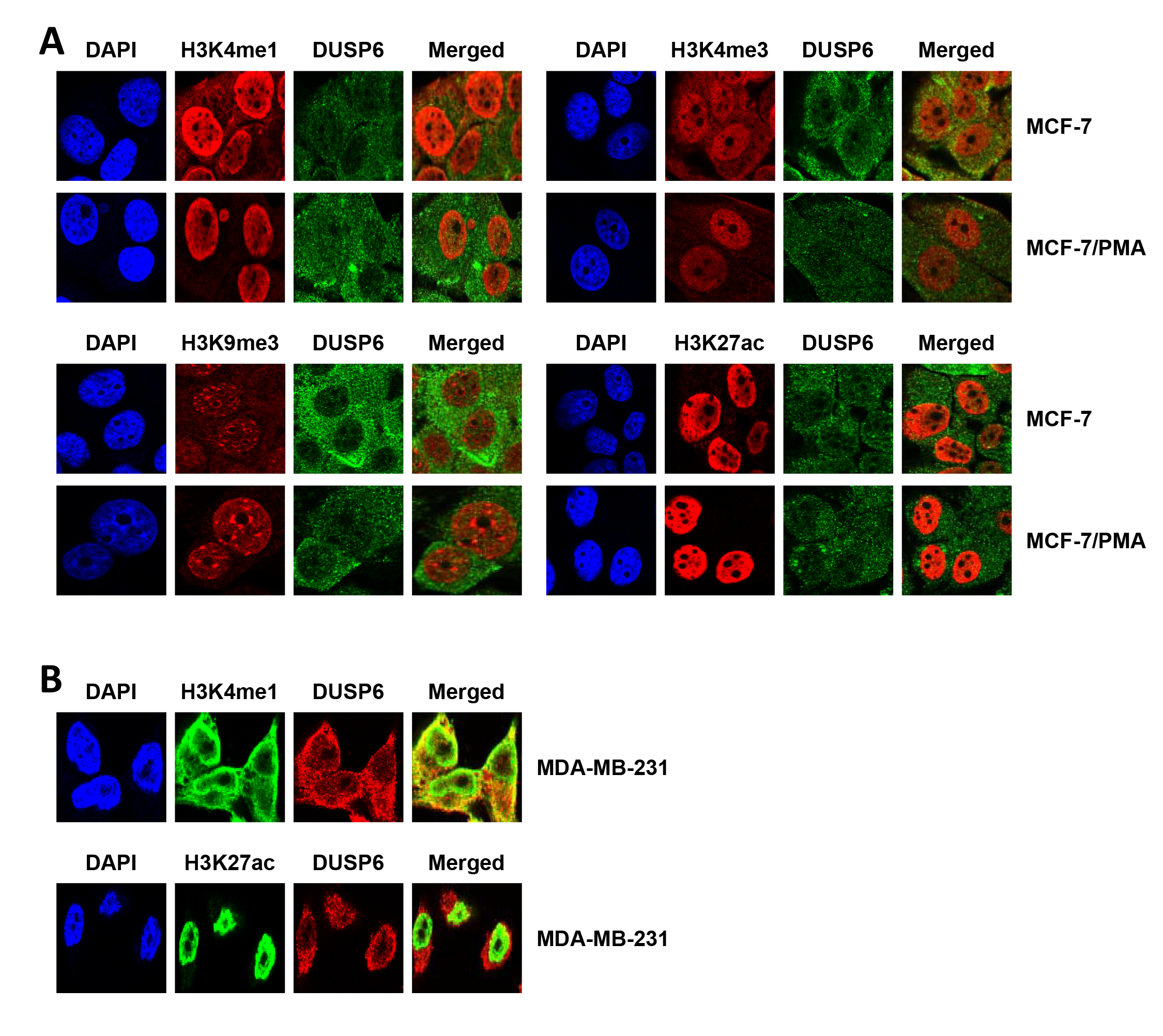

Supplement: S3 Fig — Cells were fixed and probed with primary anti-goat DUSP6 followed by anti-goat secondary antibody conjugated to Alexa-Fluor 488. (A) MCF-7 cells probed with primary anti-goat DUSP6 and either anti-rabbit H3K4me1, anti-rabbit H3K4me3, anti-rabbit H3K9me3, or anti-rabbit H3K27ac antibodies. Representative images for above dataset are shown: green = DUSPs; red = histone marks; yellow = overlap between DUSPs and histone mark fluorescence signals. (B) MDA-MB-231 cells were probed with primary anti-goat DUSP6 and either anti-rabbit H3K4me1 or anti-rabbit H3K27ac antibodies. Representative images for above dataset are shown: red = DUSPs; green = histone marks; yellow = overlap between DUSPs and histone mark fluorescence signals. (TIF) [file pone.0148065.s003.tif]

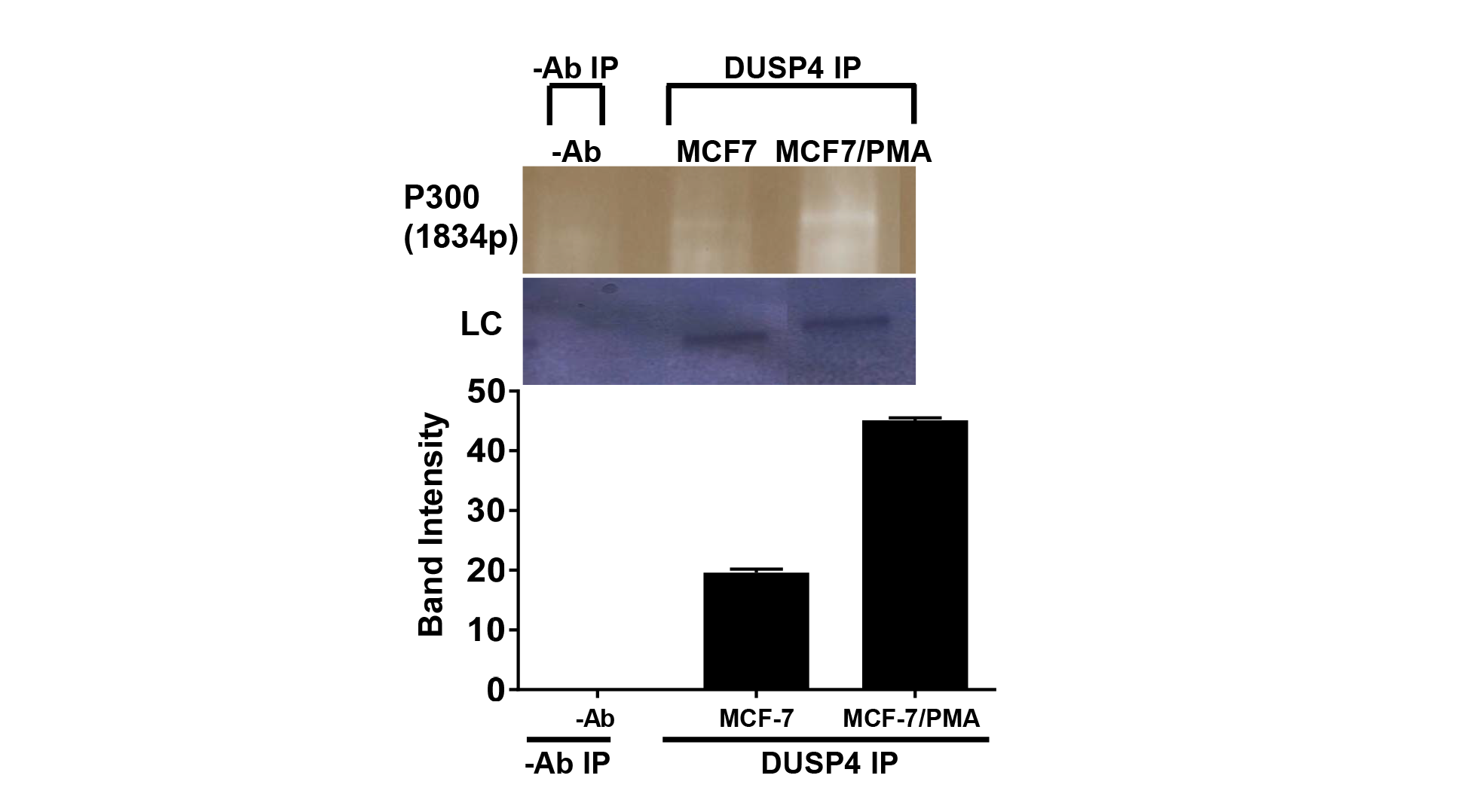

Supplement: S4 Fig — Immunoblots of the samples were probed with either a primary rabbit antibody to a phospho-epitope of p300 (p300-1834). Band intensity was plotted using ImageJ software minus background for n = 4 with mean ± SE and normalized to the LC. Representative images of the immunoblots are depicted with along with the LC. (TIF) [file pone.0148065.s004.tif]

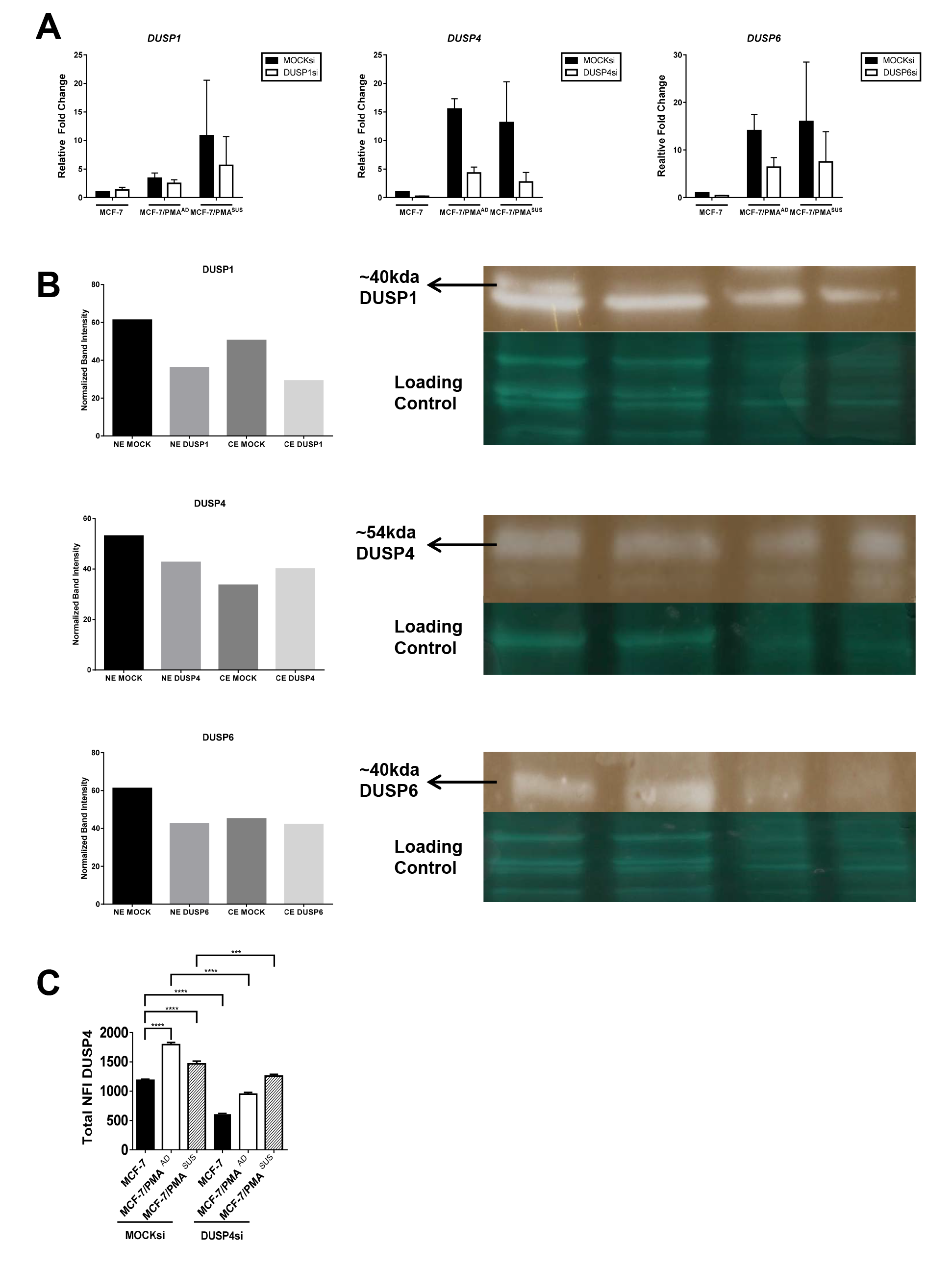

Supplement: S5 Fig — (A) Knockdown of DUSP1, DUSP4, and DUSP6 after DUSP1 (left), DUSP4 (middle), and DUSP6 (right) siRNA transfections, respectively. Transcript levels were measured by real-time PCR and data represent the mean ± SE of five independent experiments. (B) Immunoblots of MCF-7 nuclear extracts (NE) and cytoplasmic extracts (CE). Extracts were probed with primary antibodies to DUSP1, DUSP4 or DUSP6, followed by the respective HRP-conjugated secondary antibody. Signals were detected with enhanced chemiluminescence reagents, bands of the correct MW were analysed with ImageJ for intensity, and the protein loading was normalised with a total protein loading control detection kit. Graph displays the relative protein band signal intensity. (C) Confocal laser scanning microscopy was performed on MCF-7 cells were transfected with DUSP4 siRNA and subsequently incubated with either vehicle alone or PMA for 60 h. Cells were fixed and probed with primary anti-mouse DUSP4. The total nuclear intensity of DUSP4 was measured using ImageJ software to select the nucleus of each cell and measure the total NFI signal minus background for at least 20 individual cells ± SE plotted in the bar graph. (TIF) [file pone.0148065.s005.tif]
